# Enhancing nurses’ clinical decision-making confidence through dual pathways of self-directed learning: A structural equation model

**DOI:** 10.1371/journal.pone.0351551

**Published:** 2026-06-11

**Authors:** Liya Wu, Mengyu Zhang, Juan Ye

**Affiliations:** Zhejiang University School of Medicine First Affiliated Hospital, Hangzhou, Zhejiang Province, China; School of Nursing Sao Joao de Deus, Evora University, PORTUGAL

## Abstract

**Background:**

Nurses operate in complex clinical environments demanding autonomous judgment, emotional regulation, and interpersonal collaboration, yet the mechanisms through which psychological and learning resources support clinical decision-making confidence remain inadequately characterized.

**Objective:**

To examine the dual mediating roles of cognitive and relational self-directed learning in the relationship between emotional intelligence and clinical decision-making confidence among registered nurses.

**Design:**

**Cross-sectional survey study.:**

**Setting:**

Multiple medical schools and affiliated teaching hospitals across Eastern, Central, and Western China.

**Participants:**

1,478 registered nurses recruited via convenience sampling between June and August 2025.

**Methods:**

Emotional intelligence was assessed using the Wong and Law Emotional Intelligence Scale, self-directed learning via a reorganized two-factor version of the Self-Rating Scale of Self-Directed Learning, and clinical decision-making confidence via the Clinical Decision-Making Self-Confidence Scale. Structural equation modeling with bias-corrected bootstrapping (5,000 resamples) tested the mediation hypotheses.

**Results:**

Emotional intelligence significantly predicted both cognitive self-directed learning (β = 0.643, *p* < 0.01) and relational self-directed learning (β = 0.601, *p* < 0.01), as well as clinical decision-making confidence in both models. In the model with cognitive self-directed learning as mediator, the indirect effect was 0.339 (95% CI [0.273, 0.409]), representing 71.8% of the total standardized effect. In the separate model with relational self-directed learning as mediator, the indirect effect was 0.241 (95% CI [0.185, 0.302]), representing 50.8% of the total standardized effect. Both pathways were statistically significant.

**Conclusions:**

Both cognitive and relational self-directed learning are significant mediators of the relationship between emotional intelligence and clinical decision-making confidence. These findings support integrating emotional intelligence training with self-directed learning development in nursing education programs.

## 1 Introduction

Contemporary healthcare systems place unprecedented demands on nursing professionals, requiring autonomous clinical reasoning and independent judgment in increasingly complex patient care scenarios [1]. These environments necessitate rapid assessment, critical thinking, and therapeutic intervention while managing emotional stressors and interpersonal dynamics within healthcare teams [2]. Modern nursing practice emphasizes evidence-based decision-making, collaborative care coordination, and adaptive problem-solving that extend beyond traditional task-oriented responsibilities [3]. Such demands intensify in resource-constrained or high-pressure settings, where adequate psychological resources and supportive practice environments prove essential for sustaining professional performance quality [4]. Maintaining composure while making sound clinical judgments has accordingly emerged as a fundamental competency determining both patient outcomes and professional satisfaction within nursing practice.

Emotional Intelligence (EI) encompasses the ability to recognize, understand, and effectively manage both personal emotions and those of others within professional contexts. This multidimensional construct incorporates self-emotional appraisal, others’ emotional appraisal, emotional regulation, and emotional utilization as core components influencing interpersonal effectiveness and professional performance [5]. Self-Directed Learning (SDL) represents an individual’s capacity to take initiative in diagnosing learning needs, formulating goals, identifying resources, implementing strategies, and evaluating outcomes independently [6]. Within nursing contexts, SDL manifests through cognitive processes such as metacognitive awareness and learning strategy implementation, alongside social dimensions including collaborative learning and mentorship relationships. Clinical Decision-Making Confidence (CDMC) reflects a nurse’s self-assurance in identifying patient problems, interpreting clinical data, implementing appropriate interventions, and evaluating care effectiveness. Nursing self-concept, understood as the psychological sense of professional identity and capability, constitutes a core predictor of such clinical decision-making performance [7]. Despite growing recognition of these constructs’ individual significance, existing scholarship has predominantly examined self-directed learning as a unified construct, with limited investigation of how its cognitive and relational dimensions function as distinct mediating pathways in the development of clinical decision-making confidence. Therefore, this study addresses this gap by examining cognitive and relational self-directed learning as analytically distinct mediating pathways through which emotional intelligence is associated with clinical decision-making confidence.

Emotional intelligence provides the psychological foundation for managing stress and uncertainty inherent in clinical environments, potentially facilitating both cognitive and social learning processes. Enhanced emotional capabilities may enable nurses to engage more effectively in self-directed learning through improved motivation, self-regulation, and interpersonal collaboration, with the knowledge and professional networks thus cultivated potentially associated with clinical competence and decision-making capability. This suggests that emotional intelligence may exert both direct effects on clinical confidence and indirect effects mediated through self-directed learning mechanisms.

Building on these theoretical foundations, this study investigates the relationships between emotional intelligence, self-directed learning, and clinical decision-making confidence among registered nurses through three objectives:

**Objective 1:** Examine associations between demographic characteristics and clinical decision-making confidence levels among registered nurses.

**Objective 2:** Investigate the dual mediation pathways of self-directed learning in the relationship between emotional intelligence and clinical decision-making confidence, specifically examining both cognitive and relational self-directed learning mechanisms.

**Objective 3:** Compare the mediating strength of cognitive self-directed learning versus relational self-directed learning to identify which specific pathway most effectively mediates the relationship between emotional intelligence and clinical decision-making confidence.

**Fig 1:**
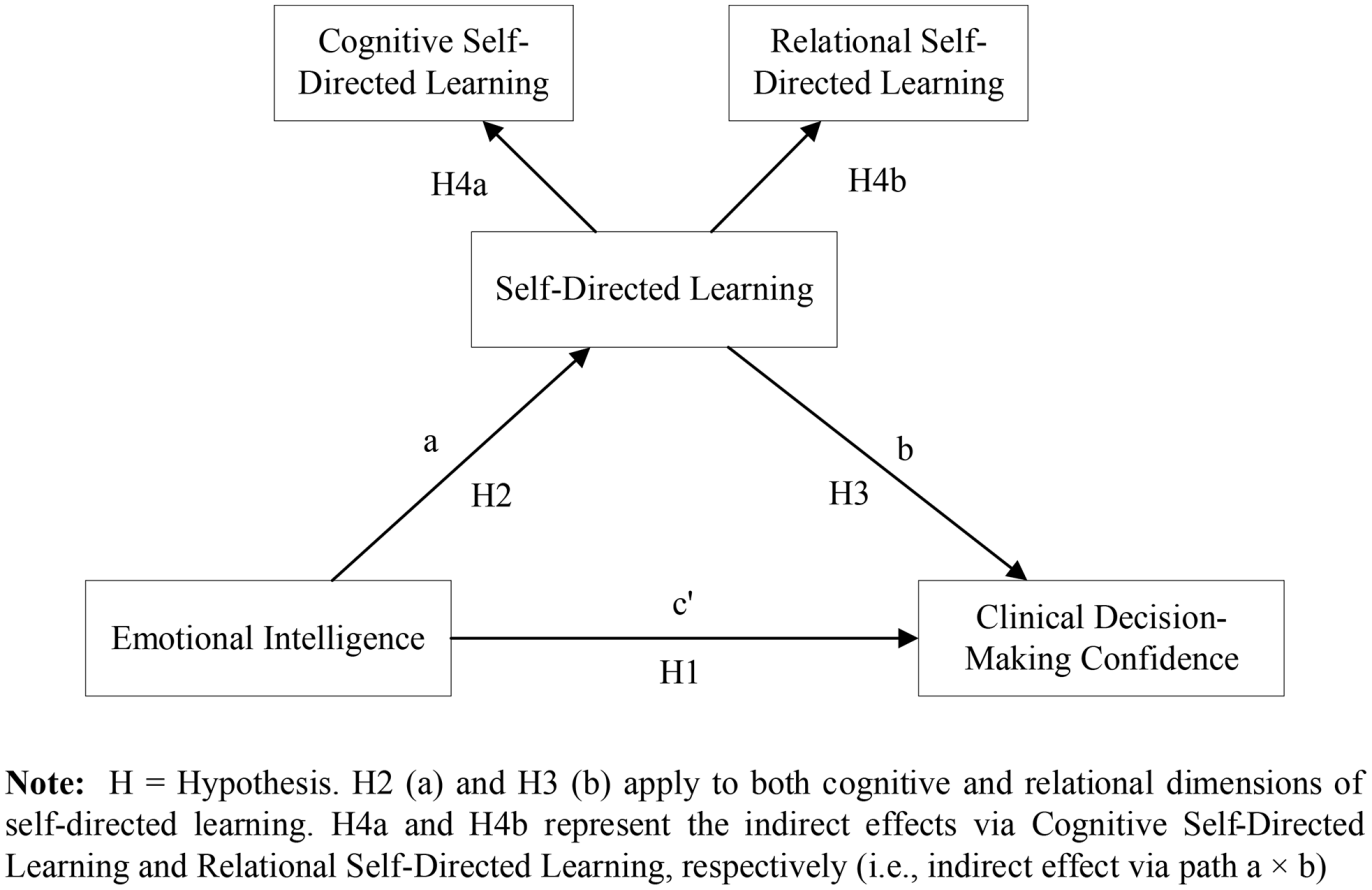
Model Hypothesis Diagram.

Building on our theoretical framework (Fig 1), we develop the following hypotheses:

Emotional intelligence is positively associated with clinical decision-making confidence, potentially through superior stress management and emotional regulation capabilities [8]. Emotionally intelligent practitioners exhibit heightened self-awareness regarding their emotional responses to complex clinical situations and enhanced ability to regulate these emotions while maintaining focus on patient safety. Their capacity to perceive and interpret emotional cues from patients, families, and healthcare team members may be associated with more effective collaborative decision-making [9], and their facility in harnessing emotional information for problem-solving may support cognitive processing during high-stakes clinical scenarios.

***Hypothesis 1 (c’):*** *Emotional intelligence positively predicts clinical decision-making confidence.*

Emotional intelligence may serve as a significant predictor of engagement in self-directed learning within nursing practice. Through emotional regulation, high-EI nurses may reduce the cognitive load imposed by demanding clinical environments, potentially preserving the psychological resources necessary for systematic knowledge acquisition and critical analysis [10]. Their heightened self-awareness may facilitate accurate identification of personal learning needs and knowledge gaps, while the interpersonal dimensions of emotional intelligence may support collaborative learning experiences and mentorship relationships that characterize effective self-directed learning in clinical settings.

***Hypothesis 2 (a):*** *Emotional intelligence positively predicts self-directed learning.*

Self-directed learning is positively associated with clinical decision-making confidence, potentially through knowledge acquisition, skill development, and professional competence advancement. Nurses who actively engage in autonomous learning demonstrate expanded clinical knowledge bases providing comprehensive foundations for evidence-based decision-making across diverse patient care scenarios [11]. The metacognitive skills and self-regulation abilities cultivated through self-directed learning may be associated with enhanced professional self-efficacy and confidence when implementing nursing interventions [12].

***Hypothesis 3 (b):*** *Self-directed learning positively predicts clinical decision-making confidence.*

Drawing from Self-Determination Theory and Experiential Learning Theory, this study establishes a theoretical framework examining the mediation of self-directed learning in the relationship between emotional intelligence and clinical decision-making confidence. Self-Determination Theory emphasizes that psychological well-being emerges through satisfaction of autonomy, competence, and relatedness [13], needs that emotional intelligence may facilitate through enhanced self-regulation and interpersonal connection. Of particular relevance to the relational mediation pathway, satisfaction of the relatedness need through emotionally intelligent professional engagement may strengthen autonomous motivation for collaborative and observationally based learning, constituting the psychological foundation of relational self-directed learning behaviors. High-EI nurses may further translate this interpersonal orientation into sustained work engagement, which could provide the motivational continuity necessary for participation in social and observational learning processes [14]. Experiential Learning Theory further positions emotional intelligence as potentially associated with reflective observation and abstract conceptualization within learning cycles; interpersonal exchange and peer dialogue may serve as primary vehicles for this reflective process, potentially enabling deeper processing of clinical experiences, and high-EI practitioners may be better positioned to leverage such exchanges as mechanisms for knowledge integration, with colleagues potentially serving as social learning resources through whom experiential clinical knowledge may be actively acquired and refined [15]. Informed by insights from Yue et al. [16] regarding this conceptual distinction, this study treats cognitive and relational self-directed learning as two analytically separable mediating pathways rather than a unified construct.

Within nursing education contexts, emotional intelligence may be associated with conditions conducive to autonomous learning engagement, which in turn may contribute to the competence foundation necessary for confident clinical decision-making. While prior research has broadly examined self-directed learning as a unified construct, systematic empirical investigation of its cognitive and relational dimensions as independent mediating pathways remains limited. The following hypotheses therefore represent an exploratory extension of existing frameworks.

***Hypothesis 4a (a × b):*** *Cognitive self-directed learning mediates the relationship between emotional intelligence and clinical decision-making confidence.*

***Hypothesis 4b (a × b):*** *Relational self-directed learning mediates the relationship between emotional intelligence and clinical decision-making confidence.*

These investigations will inform targeted educational interventions that strengthen critical professional capabilities in complex healthcare environments.

## 2 Methodology

### 2.1 Aim

This study examined whether self-directed learning mediates the relationship between emotional intelligence and clinical decision-making confidence among Chinese nursing professionals in hospital settings.

### 2.2 Participants, design and setting

This cross-sectional survey study employed convenience sampling, which offers practical advantages in recruitment efficiency and feasibility, though it is subject to potential selection bias that may limit the generalizability of findings [17]. Following the STROBE guidelines for cross-sectional studies (S1 Checklist), between June and August 2025, questionnaires were distributed to registered nurses across multiple Chinese medical schools and affiliated teaching hospitals.

The study was conducted across multiple medical institutions in Eastern, Central, and Western China, encompassing both top-tier hospitals and provincial medical centers. This institutional diversity ensured representation of varied clinical environments and professional development resources available to Chinese nurses. Prior to study initiation, permission was secured from all participating institutions. The recruitment process followed a standardized protocol across all participating institutions. Nursing department coordinators at each institution assisted in distributing study information through official channels. Potential participants were approached during regular shift changes or scheduled breaks, ensuring minimal disruption to their clinical responsibilities.

Inclusion criteria were: (1) Currently employed as a registered nurse; (2) Minimum of one-year clinical nursing experience; (3) Voluntary participation with informed consent; (4) Currently working in mainland China with proficiency in reading and writing Chinese language. Exclusion criteria were: (1) Nursing students or nursing interns; (2) Administrative staff without direct patient care responsibilities; (3) Nurses on extended leave (maternity, medical, or sabbatical leave exceeding three months); (4) Part-time or temporary contract nurses with less than six months of tenure at the current institution.

### 2.3 Sample size

Sample size determination utilized Mardia’s statistical guidelines, which emphasize the importance of adequate participant-to-variable ratios in multivariate analyses. Following the established convention of recruiting 5–10 participants per measured variable to ensure sufficient statistical power [18], our study calculated requirements based on 13 variables within the measurement framework. This yielded a baseline range of 65–130 participants as the fundamental sample requirement. To accommodate potential data attrition and response quality concerns, a 20% buffer was incorporated, establishing the minimum threshold at 156 participants. The actual sample obtained considerably exceeded this benchmark, ensuring robust analytical capacity for all statistical procedures.

### 2.4 Measures

Data collection involved three established instruments to assess the primary study variables: emotional intelligence, self-directed learning, and clinical decision-making confidence. Each measurement tool was obtained from peer-reviewed publications, with appropriate citations documented in the reference section.

#### 2.4.1 General information questionnaire.

A self-developed demographic questionnaire was constructed following established guidelines for cross-sectional survey instrument design [19]. The questionnaire captured participant characteristics across four key domains: (1) personal demographics (age, gender, marital status), (2) professional credentials (educational attainment, nursing rank), (3) workplace characteristics (hospital classification, clinical unit), and (4) scholarly engagement (research involvement, publication record, conference attendance). Supplementary data encompassed employment status, clinical experience duration, and household income per capita.

#### 2.4.2 Wong and law emotional intelligence scale (WLEIS).

Emotional intelligence assessment utilized the WLEIS, originally developed by Wong and Law [5]. This 16-item instrument evaluates four core competencies: self-emotion appraisal (recognizing personal emotional states), others’ emotion appraisal (understanding emotional cues from colleagues), emotion regulation (managing emotional responses), and emotion utilization (applying emotional awareness to enhance problem-solving). Responses are captured using a 7-point Likert format. The Chinese adaptation by Wang [20] has demonstrated robust psychometric properties. In the present investigation, the scale exhibited strong internal consistency (Cronbach’s α = 0.843) and adequate model fit in confirmatory factor analysis (χ²/df = 2.543, CFI = 0.966, TLI = 0.958, RMSEA = 0.039, SRMR = 0.035). Standardized factor loadings ranged from 0.57 to 0.79, with composite reliability (CR) between 0.69–0.80 and average variance extracted (AVE) between 0.36–0.49 (S2 Table).

#### 2.4.3 Self-rating scale of self-directed learning (SRSSDL).

The assessment of self-directed learning employed Williamson’s SRSSDL [6], a 60-item instrument measured on a 5-point Likert scale encompassing five dimensions of learner autonomy: Learning Awareness, Learning Strategies, Learning Behavior, Learning Evaluation, and Interpersonal Relationships. Shen and Hu’s Chinese adaptation has established strong psychometric credentials across diverse populations [21].

Inspired by the dimensional distinctions suggested by Yue et al. [16], the five original dimensions were reorganized into two theoretically distinct subscales. The Cognitive Self-Directed Learning subscale (C-SDL) comprises 48 items drawn from Learning Awareness, Learning Strategies, Learning Behavior, and Learning Evaluation, capturing internal cognitive and self-regulatory processes of autonomous learning. The Social and Relational Self-Directed Learning subscale (SR-SDL) comprises the 12 items from the Interpersonal Relationships dimension, reflecting collaborative and social mechanisms supporting autonomous professional development; in subsequent analyses, “Interpersonal Relationships” serves as its operational indicator. The scale demonstrated strong internal consistency (Cronbach’s α = 0.937), and confirmatory factor analysis yielded acceptable fit for the two-factor structure (χ²/df = 2.709, CFI = 0.803, TLI = 0.796, RMSEA = 0.041, SRMR = 0.043). Although comparative CFA indicated that the original five-factor structure yielded superior statistical fit (Δχ² = 796.09, Δdf = 9, *p* < 0.01), the two-factor reorganization was retained given its conceptual alignment with this distinction, with absolute fit indices remaining within accepted thresholds. Factor loadings ranged from 0.279 to 0.616, with CR values of 0.736–0.838 and AVE values of 0.190–0.306 (S2 Table); constructs with AVE below 0.50 were retained to preserve theoretical completeness [22].

#### 2.4.4 Clinical decision-making self-confidence scale (CDMSCS).

Clinical decision-making confidence was measured using Hart and colleagues’ CDMSCS, a 12-item instrument designed to evaluate nurses’ perceived competence in managing acute patient deterioration scenarios [23]. The scale employs a 5-point Likert response format and comprises two subscales: respiratory/cardiac events (8 items) and neurological events (4 items), collectively explaining 73.81% of variance. The original validation demonstrated excellent psychometric properties with alpha coefficients between 0.83–0.98. Translation into Chinese followed standard forward-backward procedures with expert review and pilot validation. In the present investigation, the scale exhibited strong internal consistency (Cronbach’s α = 0.821) and adequate model fit in confirmatory factor analysis (χ²/df = 5.377, CFI = 0.919, TLI = 0.889, RMSEA = 0.065, SRMR = 0.049). The CFA results for the full sample yielded factor loadings between 0.49–0.71, CR values between 0.57–0.66, and AVE values between 0.30–0.40 (S2 Table).

### 2.5 Data collection

**Fig 2:**
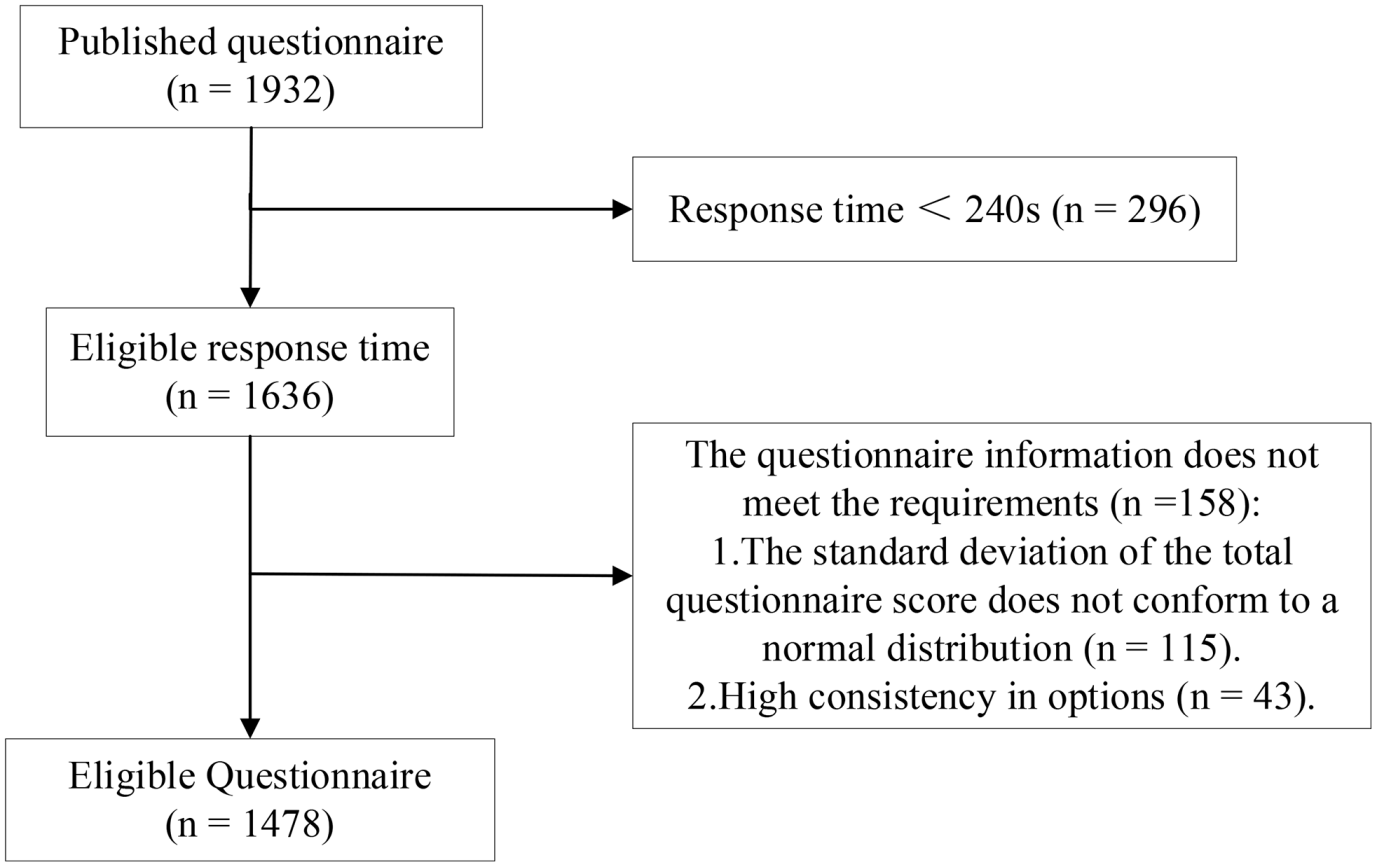
The Process of Data Exclusion.

Prior to survey administration, research personnel underwent comprehensive standardized training protocols. The Questionnaire Star platform facilitated questionnaire distribution, accompanied by detailed participant instructions. Data integrity measures included IP address restrictions preventing multiple submissions from identical sources, alongside a one-hour completion window for all responses. Two independent researchers conducted systematic data review, implementing rigorous evidence-based exclusion criteria to safeguard quality and reliability: (1) Questionnaires completed in less than 240 seconds represented unrealistic completion times, with this threshold derived from researcher pilot testing and validated by Huang et al.’s methodology for detecting insufficient effort responses [24]; (2) Questionnaires requiring more than one hour for completion, consistent with established protocols for identifying inadequate engagement patterns; (3) Response patterns demonstrating excessive regularity, specifically selecting identical options across 10 or more consecutive items, following Schell’s criteria for detecting systematic response bias [25]; (4) Responses falling beyond two standard deviations from the mean were eliminated to maintain data quality standards, as recommended by Van Quaquebeke [26]. From 1932 distributed questionnaires, 1478 valid responses were obtained, yielding an effective response rate of 76.5%. Fig 2 depicts the systematic data exclusion process.

### 2.6 Data analysis

Data analysis employed a multi-software approach utilizing Microsoft Excel, IBM SPSS Statistics v.26, R, and Amos to ensure comprehensive evaluation of study findings. Following Collier’s structural equation modeling framework [27], preliminary data screening examined missing values, outliers, and fundamental assumptions including normality, linearity, and multicollinearity according to Field’s recommendations [28]. Reliability and validity assessments were conducted using R. Common method bias was evaluated using common method factor (CMF) analysis in R, whereby an unmeasured latent common method factor was modeled alongside all substantive constructs [29]. Descriptive statistics, t-tests, ANOVA, and post hoc analyses facilitated group comparisons, while one-way ANOVA with Bonferroni corrections examined demographic group differences. Pearson correlation and stepwise linear regression identified predictors of clinical decision-making confidence.

Structural equation modeling (SEM) utilizing Amos examined hypothesized relationships between emotional intelligence, self-directed learning (cognitive and relational), and clinical decision-making confidence following Collier’s methodology. Model fit evaluation incorporated multiple indices: χ2/df ratio (<3.0), GFI (>0.90), AGFI (>0.90), CFI (>0.90), and RMSEA (<0.08) [27]. Given the well-documented sensitivity of the χ2/df statistic to large sample sizes, a relaxed threshold of <5.0 was considered acceptable in the present study in accordance with established guidelines [30]. Mediating effects underwent assessment through bootstrapping procedures with 5,000 random samples to ensure analytical robustness. Statistical significance for mediation was established when bias-corrected confidence intervals excluded zero, confirming reliable pathways between emotional intelligence and clinical decision-making confidence. All statistical procedures employed two-tailed testing with significance thresholds set at *p* < 0.05.

### 2.7 Ethical considerations

The study received ethical approval in June from the Clinical Research Ethics Committee of the First Affiliated Hospital, Zhejiang University School of Medicine (Approval number: IIT20250895B-R2). The research adhered to the principles of the Declaration of Helsinki and STROBE guidelines. Participants were provided with information about the study’s purpose and procedures, and all provided electronic informed consent. Confidentiality was protected through unique identifiers and secure data storage with restricted access. Participants were informed of their voluntary participation and right to withdraw at any time.

## 3 Results

The average method factor accounted for 15.03% of the total variance, falling below the recommended 25% threshold, indicating that common method bias was not a significant concern in the present study [29].

### 3.1 Participants’ sociodemographic characteristics

Among the 1478 participants, female nurses comprised 97.56% of the sample, with the largest age group being 30–40 years (40.87%). Most participants were married (73.41%), held bachelor’s degrees (92.29%), worked in tertiary hospitals (97.70%), and held permanent employment status (68.07%). Univariate analysis demonstrated significant associations between Clinical Decision-Making Self-Confidence Scale scores and eight demographic factors (all *p* < 0.01): age, marital status, job title, research project participation, article publication, academic conference participation, employment type, and years of work experience. Post-hoc analyses (S3 Table) revealed specific between-group differences for these demographic variables. Table 1 provides comprehensive statistical analyses for these findings.

**Table 1:** Demographic Data and Univariate Analysis (n = 1478).

Variable	Frequency (%)	Clinical Decision-Making Self-Confidence Scale	*t/F*	*p* value
**Age**				
20-30 years	359 (24.29)	44.83 ± 6.54	20.30	<0.01**
30-40 years	604 (40.87)	47.23 ± 6.82		
40-50 years	384 (25.98)	48.37 ± 6.25		
＞50 years	131 (8.86)	48.16 ± 6.32		
**Gender**				
Male	36 (2.44)	44.94 ± 6.98	−1.89	0.06
Female	1442 (97.56)	47.08 ± 6.68		
**Marital Status**				
Single	358 (24.22)	45.17 ± 6.25	18.67	<0.01**
Married	1085 (73.41)	47.61 ± 6.72		
Divorced or Widowed	35 (2.37)	47.86 ± 6.83		
**Education Level**				
Associate degree or below	71 (4.80)	44.83 ± 5.57	1.39	0.25
Bachelor’s degree	1364 (92.29)	46.07 ± 5.71		
Master’s degree or above	43 (2.91)	47.26 ± 5.68		
**Household monthly income per capita**				
<5000	119 (8.05)	44.64 ± 5.97	2.16	0.09
5000-8000	536 (36.27)	45.78 ± 6.14		
8000-10000	356 (24.09)	47.12 ± 6.05		
>10000	467 (31.60)	48.46 ± 6.33		
**Job Title**				
Nurse	128 (8.66)	44.06 ± 7.01	19.13	<0.01**
Senior Nurse	479 (32.41)	46.33 ± 6.50		
Charge Nurse	724 (48.99)	47.49 ± 6.59		
Deputy Chief or Chief Nurse	147 (9.95)	49.59 ± 6.34		
**Hospital Level**				
Tertiary Hospital	1444 (97.70)	47.02 ± 6.61	−0.03	0.98
Secondary Hospital	34 (2.30)	47.06 ± 9.48		
**Research Project Participation Experience**				
None	1098 (74.29)	46.50 ± 6.65	−5.16	<0.01**
Yes	380 (25.71)	48.54 ± 6.57		
**Article Publication Experience**				
None	627 (42.42)	45.81 ± 6.67	−6.07	<0.01**
Yes	851 (57.58)	47.92 ± 6.57		
**Academic Conference Participation Experience**				
None	654 (44.25)	46.07 ± 6.89	−4.95	<0.01**
Yes	824 (55.75)	47.79 ± 6.43		
**Employment Type**				
Labor Dispatch	261 (17.66)	45.53 ± 6.03	8.28	<0.01**
Contract	211 (14.28)	47.67 ± 7.05		
Permanent	1006 (68.07)	47.28 ± 6.73		
**Years of Work Experience**				
1-5 years	267 (18.07)	44.26 ± 6.18	20.84	<0.01**
6-10 years	245 (16.58)	46.97 ± 6.38		
11-15 years	390 (26.39)	47.20 ± 7.30		
16-20 years	240 (16.24)	46.93 ± 5.73		
＞20 years	336 (22.73)	49.13 ± 6.43		
**Department**				
Internal Medicine	486 (32.88)	48.59 ± 5.72	2.38	0.02*
Surgery	405 (27.40)	47.85 ± 5.38		
ICU	61 (4.13)	48.15 ± 5.66		
Emergency	54 (3.65)	46.52 ± 5.17		
Operating Room	120 (8.12)	45.53 ± 5.49		
Pediatrics	26 (1.76)	44.81 ± 5.08		
Gynecology	26 (1.76)	47.23 ± 5.57		
Obstetrics	65 (4.40)	42.97 ± 6.19		
Other	235 (15.90)	46.83 ± 5.22		

*p < 0.05; **p < 0.01.

### 3.2 Descriptive statistics and correlation analysis

Psychological scale assessments revealed moderate to high performance levels across all measures. On the Wong and Law Emotional Intelligence Scale, item-average scores across subscales ranged from 5.16 ± 1.12 (Regulation of Emotion) to 6.12 ± 0.87 (Self-Emotion Appraisal) on a 7-point scale. The Clinical Decision-Making Self-Confidence Scale yielded item-average subscale scores ranging from 3.90 ± 0.53 (Evaluation) to 4.08 ± 0.57 (Identification) on a 5-point scale, indicating moderate to moderately high clinical decision-making self-confidence. Item-average subscale scores on the Self-Rating Scale of Self-Directed Learning ranged from 3.81 ± 0.53 (Learning Behavior) to 4.01 ± 0.41 (Learning Awareness) on a 5-point scale. Correlation analysis among all 16 variables demonstrated strong positive relationships throughout the dataset (all *p* < 0.01). Comprehensive descriptive statistics and correlation coefficients are documented in S4 Table.

### 3.3 Multiple linear regression of clinical decision-making confidence in nurses

Multiple linear regression analysis identified a model accounting for 38.9% of variance in clinical decision-making confidence (F = 79.752, *p* < 0.01, adjusted R² = 0.384), with six significant predictors (all *p* < 0.01), as detailed in Table 2. Learning awareness was the most influential factor (β = 0.226), followed by learning behavior (β = 0.202), years of work experience (β = 0.167), interpersonal relationships (β = 0.150), and use of emotion (β = 0.095). Department affiliation demonstrated a negative association with confidence levels (β=−0.104). VIF values ranged from 1.032 to 2.928, confirming the absence of multicollinearity.

**Table 2:** Stepwise linear regression of clinical decision-making confidence in nurses (n = 1478).

Items	Regression Coefficient	Standard Error	Standardized Regression Coefficient	t	*p* Value	Covariance Tolerance	VIF
(Constant)	15.211	1.652		9.208	<0.01**		
Learning Awareness	0.262	0.049	0.226	5.366	<0.01**	0.46	2.176
Learning Behavior	0.182	0.042	0.202	4.346	<0.01**	0.375	2.666
Years of Work Experience	0.695	0.122	0.167	5.698	<0.01**	0.948	1.054
Interpersonal Relationships	0.144	0.047	0.15	3.079	<0.01**	0.341	2.928
Department	−0.203	0.057	−0.104	−3.577	<0.01**	0.969	1.032
Use of Emotion	0.14	0.052	0.095	2.693	<0.01**	0.652	1.533

*R*² = 0.389, adjust *R*² = 0.384, *F* = 79.752, *p < 0.05; **p < 0.01.

### 3.4 Path analysis

#### 3.4.1 Pathway analysis of clinical decision-making confidence in nurses mediated by cognitive self-directed learning.

**Fig 3:**
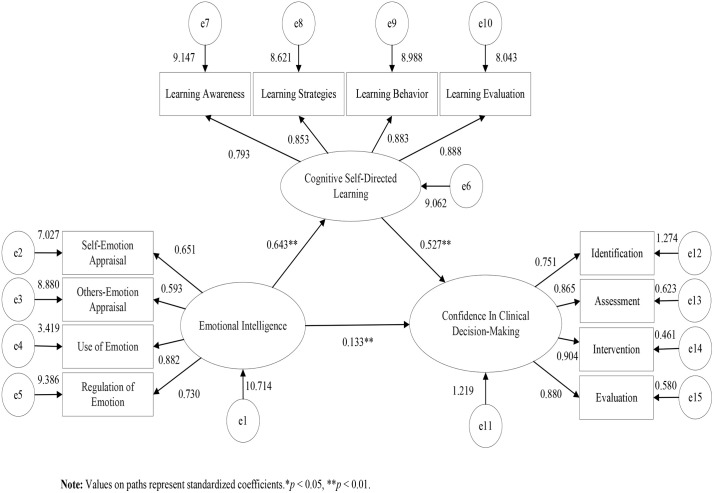
Structural equation model of cognitive self-directed learning in emotional intelligence and clinical decision-making confidence in nurses (n = 1478).

The structural equation model demonstrated excellent fit indices (CMIN/DF = 4.475, GFI = 0.949, AGFI = 0.922, CFI = 0.971, RMSEA = 0.068), as presented in Fig 3. Emotional intelligence was positively associated with cognitive self-directed learning (*β* = 0.643, *p* < 0.01) and with clinical decision-making confidence (*β* = 0.133, *p* < 0.01). Cognitive self-directed learning also positively affected clinical decision-making confidence (*β* = 0.527, *p* < 0.01).

#### 3.4.2 Pathway analysis of clinical decision-making confidence in nurses mediated by relational self-directed learning.

**Fig 4:**
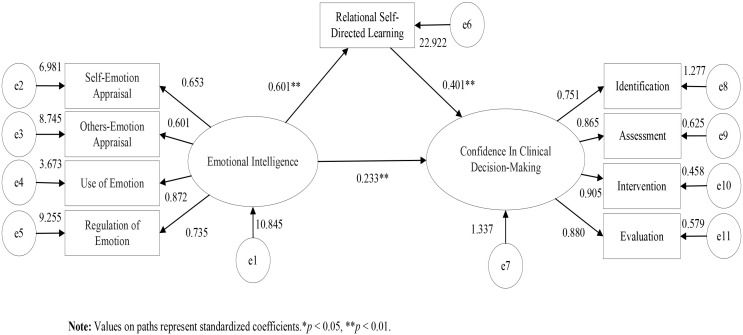
Structural equation model of relational self-directed learning in emotional intelligence and clinical decision-making confidence in nurses (n = 1478).

The structural equation model exhibited excellent fit parameters (CMIN/DF = 4.285, GFI = 0.951, AGFI = 0.925, CFI = 0.973, RMSEA = 0.066), as depicted in Fig 4. Emotional intelligence demonstrated a robust positive relationship with relational self-directed learning (*β* = 0.601, *p* < 0.01) and maintained a direct positive association with clinical decision-making confidence (*β* = 0.233, *p* < 0.01). Relational self-directed learning also significantly enhanced clinical decision-making confidence (*β* = 0.401, *p* < 0.01).

### 3.5 Bootstrap analysis

Bias-corrected bootstrap analysis with 5,000 resamples examined the mediating roles of cognitive and relational self-directed learning (Table 3). Cognitive self-directed learning (Model 1) mediated 71.8% of the total standardized effect of emotional intelligence on clinical decision-making confidence (indirect effect = 0.339, 95% CI [0.273, 0.409]; total effect = 0.472, 95% CI [0.403, 0.536]). Relational self-directed learning (Model 2) accounted for 50.8% of the total standardized effect (indirect effect = 0.241, 95% CI [0.185, 0.302]; total effect = 0.474, 95% CI [0.404, 0.537]). Both mediation pathways were significant, with bias-corrected confidence intervals excluding zero.

**Table 3:** Bootstrap Test of mediation effect (n = 1478).

Model	Pathway	SE	Estimate	Lower	Upper	*p* Value
**Model 1**	Emotional Intelligence → Cognitive Self-Directed Learning	0.028	0.643	0.585	0.698	<0.01**
	Cognitive Self-Directed Learning → Confidence In Clinical Decision-Making	0.047	0.527	0.431	0.617	<0.01**
	Emotional Intelligence → Confidence In Clinical Decision-Making	0.049	0.133	0.036	0.231	0.01*
	Emotional Intelligence → Cognitive Self-Directed Learning → Confidence In Clinical Decision-Making	0.034	0.339	0.273	0.409	<0.01**
	Total	0.034	0.472	0.403	0.536	<0.01**
**Model 2**	Emotional Intelligence → Relational Self-Directed Learning	0.028	0.601	0.543	0.655	<0.01**
	Relational Self-Directed Learning → Confidence In Clinical Decision-Making	0.045	0.401	0.311	0.49	<0.01**
	Emotional Intelligence → Confidence In Clinical Decision-Making	0.049	0.233	0.133	0.326	<0.01**
	Emotional Intelligence → Relational Self-Directed Learning → Confidence In Clinical Decision-Making	0.03	0.241	0.185	0.302	<0.01**
	Total	0.034	0.474	0.404	0.537	<0.01**

*p < 0.05; **p < 0.01.

## 4 Discussion

### 4.1 Predictors of nurses’ clinical decision-making confidence

The stepwise regression analysis revealed that six variables significantly predicted nurses’ clinical decision-making confidence, collectively explaining 38.9% of the variance (*R²* = 0.389).

#### 4.1.1 Learning awareness, learning behavior, and interpersonal relationships.

The three self-directed learning dimensions identified in this study collectively underscore the fundamental importance of autonomous learning capabilities in developing clinical decision-making confidence. Learning awareness emerged as the strongest predictor among these dimensions, suggesting that metacognitive skills may function not merely as an instrumental gateway to knowledge acquisition, but as a constitutive dimension of nurses’ professional identity. Nurses with heightened awareness of their own learning processes may be better positioned to consolidate a coherent sense of professional self-concept — encompassing leadership orientation and communicative competence — that is associated with greater assurance in clinical decision-making [7]. This psychological dimension of self-directed learning, through which nurses construct and reinforce their professional identity rather than simply accumulating clinical information, may more robustly predict decision-making confidence than knowledge acquisition alone. The substantial influence of learning behavior further indicates that active engagement in autonomous learning may create a positive feedback loop associated with clinical confidence, as nurses who consistently pursue learning opportunities may develop broader knowledge bases and more sophisticated clinical reasoning capabilities that are associated with greater assurance when navigating complex decisions [31]. The significant role of interpersonal relationships suggests how social learning networks may amplify individual confidence through collective knowledge and mutual support; nurses who cultivate strong professional relationships may gain access to diverse perspectives and collaborative problem-solving opportunities that could buffer the stress associated with difficult clinical decisions, suggesting that interpersonal skills may be essential for accessing the collective wisdom necessary for confident decision-making [32].

#### 4.1.2 Use of emotion.

The significant predictive effect of use of emotion suggests that nurses’ capacity to harness emotional information for decision-making may function as a cognitive resource in complex clinical environments. Nurses who utilize emotional information effectively may demonstrate not only enhanced situational awareness but also elevated work engagement — characterized by vigor and dedication — that constitutes an affective foundation for confident clinical performance [14]. By channeling emotional information into sustained professional investment rather than allowing it to become a source of distress, nurses may be better equipped to maintain the attentional focus and motivational continuity necessary for navigating high-stakes decision-making contexts. This emotional-cognitive integration may help explain why stronger emotional utilization capabilities are associated with greater confidence in uncertain clinical situations, as both analytical and intuitive information sources can be drawn upon to inform decisions [33].

#### 4.1.3 Work experience and department.

The association between work experience and clinical decision-making confidence is consistent with the cumulative effect of clinical exposure, through which repeated engagement with diverse patient scenarios is associated with more refined pattern recognition and mature clinical judgment [34].

Department-based differences in confidence scores may reflect underlying variation in the degree of procedural standardization, clinical predictability, and emotional demands characteristic of different specialties [35], suggesting that structural features of the clinical environment are meaningfully associated with nurses’ decision-making confidence.

#### 4.1.4 Impact of sample homogeneity on confidence patterns.

The sample’s compositional characteristics warrant acknowledgment as potential influences on the observed patterns. The concentration of participants from tertiary hospitals (97.70%) likely reflects an environment of greater training resources and clinical complexity, which may have contributed to the elevated overall confidence scores and the significant department-based variation observed in the regression model. The predominance of female nurses (97.56%) may have similarly amplified the predictive role of emotional utilization, given documented gender-related tendencies in emotional processing and interpersonal communication [36], and whether equivalent patterns obtain in more gender-diverse or primary care samples remains to be established.

### 4.2 Self-directed learning mediates the emotional intelligence-clinical decision-making confidence relationship through dual cognitive and interpersonal pathways

The cognitive self-directed learning pathway emerges as the predominant mechanism through which emotional intelligence influences clinical decision-making confidence, demonstrating a mediation proportion of 71.8% of the total standardized effect (*β* = 0.339, *p* < 0.01).

This cognitive mediation process may suggest that emotionally intelligent nurses may possess enhanced capacity for metacognitive monitoring and strategic learning regulation, a relationship potentially explained by emotional intelligence’s association with reduced cognitive load: by attenuating the emotional interference generated by demanding clinical environments, emotional intelligence may preserve the cognitive resources necessary for critical thinking and systematic analysis, enabling more deliberate and structured learning engagement [10]. The predominance of this pathway may reflect the inherently cognitive-intensive nature of nursing practice, where complex clinical reasoning may demand sophisticated self-regulatory mechanisms associated with decision certainty. Nurses who demonstrate elevated emotional intelligence appear to draw on their emotional awareness as a foundation for systematic learning approaches through which competence may be progressively consolidated, a pattern consistent with research identifying self-directed learning as a core mechanism for professional competence development in practice-based disciplines [37].

The relational self-directed learning pathway represents a complementary yet distinct mechanism, contributing a mediation proportion of 50.8% of the total standardized effect (*β* = 0.241, *p* < 0.01) through interpersonal learning processes that leverage collaborative knowledge exchange. This relational mediation may suggest that emotionally intelligent nurses may be more likely to extract meaningful learning experiences from social interactions, potentially utilizing their enhanced empathy and communication competencies to access collective professional knowledge associated with greater individual confidence [38]. The interpersonal pathway may operate through the satisfaction of nurses’ relatedness needs within collegial relationships: structured professional collaboration — including mentorship dyads and interdisciplinary communication — may provide not only instrumental knowledge exchange but also the compassion satisfaction that buffers the moral distress accompanying high-stakes clinical decisions, thereby reinforcing decision-making confidence through relational means [4]. Nurses with high emotional intelligence may more effectively manage stress and emotions, in ways potentially associated with enhanced interpersonal skills and overall team effectiveness, while potentially facilitating multidisciplinary collaboration associated with both individual confidence and collective professional performance [39]. The relational mechanism appears particularly crucial in healthcare environments where collaborative decision-making represents a fundamental operational requirement, suggesting that social learning processes serve essential complementary functions to individual cognitive regulation.

The coexistence of these pathways illustrates the multifaceted nature of emotional intelligence’s association with professional competence, wherein individual cognitive regulation and social learning processes may create synergistic effects associated with overall clinical development. The cognitive pathway’s predominance suggests that individual regulatory competencies may constitute the primary pathway through which emotional intelligence is associated with clinical confidence, reflecting the autonomous nature of clinical decision-making where practitioners must ultimately rely on individual judgment and expertise [40]. However, the substantial contribution of the relational pathway suggests that interpersonal learning processes may not be dismissed as secondary mechanisms, particularly given healthcare’s inherently collaborative nature where peer consultation and team-based decision-making represent standard practice [32]. Nurses with diminished emotional intelligence may experience cognitive rigidity associated with excessive emotional burden when confronting clinical obstacles, potentially rendering them more vulnerable to anxiety and frustration that could compromise both individual confidence and collaborative effectiveness [41].

These dual mediation mechanisms are consistent with the theoretical framework proposed through Self-Determination Theory and Experiential Learning Theory. The cognitive and relational pathways may illustrate how emotional intelligence is associated with satisfaction of the three fundamental psychological needs identified within Self-Determination Theory, wherein cognitive self-directed learning may promote competence satisfaction through enhanced metacognitive regulation, relational self-directed learning may facilitate relatedness through collaborative learning connections, and both pathways may collectively support autonomy through self-directed learning pursuits associated with decision-making confidence [13]. Simultaneously, the findings align with Experiential Learning Theory’s cyclical framework, wherein emotional intelligence may be associated with the reflective observation and abstract conceptualization phases of learning cycles through enhanced emotional awareness that could support deeper processing of clinical experiences and more effective knowledge integration [15].

### 4.3 Practical implications

The mediation proportions identified across the two models — cognitive self-directed learning accounting for 71.8% and relational self-directed learning for 50.8% of the respective total standardized effects — suggest potential value in integrated professional development models combining emotional intelligence training with self-directed learning skill development, with possible synergistic effects on individual competency growth and collaborative team dynamics [42]. Longitudinal research is nonetheless needed to confirm causal directionality before these models are broadly implemented.

Given that cognitive self-directed learning demonstrates the stronger mediating proportion (71.8%), educational interventions should prioritize developing metacognitive awareness and learning strategy implementation capabilities that may support nurses in systematically monitoring their decision-making processes and building clinical confidence [43]. This requires integration of reflective practice methodologies, critical thinking exercises, and metacognitive training programs within both pre-licensure and continuing education curricula [43]. Simultaneously, the meaningful contribution of relational self-directed learning (50.8%) necessitates parallel development of collaborative learning competencies through structured mentorship programs, peer consultation networks, and interdisciplinary team-based learning initiatives that may leverage interpersonal relationships in supporting professional growth and confidence building [44].

The experience-based findings indicating that nurses with twenty or more years of clinical practice demonstrate superior decision-making confidence highlight opportunities for workforce development strategies. While extensive clinical experience is associated with stronger decision-making competencies, veteran nurses represent valuable mentorship resources whose experiential knowledge can be systematically leveraged through structured relational self-directed learning programs, while simultaneously requiring their own professional development support to maintain currency with evolving practice standards. Furthermore, the departmental variations in clinical decision-making confidence underscore the importance of context-specific interventions that acknowledge the unique decision-making challenges and collaborative dynamics inherent in different clinical specialties.

## 5 Limitations and prospects

This study has several limitations that warrant consideration. (1) Due to its cross-sectional design, causal inferences cannot be drawn regarding the relationships among emotional intelligence, self-directed learning, and clinical decision-making confidence. (2) The sample consisted primarily of female nurses from tertiary hospitals, which may limit the generalizability of the findings across different clinical settings, hospital levels, and genders. (3) The reliance on self-report measures introduces potential bias, including social desirability bias; in particular, clinical decision-making confidence is an inherently subjective construct that was not corroborated by objective clinical performance indicators.

Future research should address these limitations by (1) adopting longitudinal or quasi-experimental designs to explore causal mechanisms, (2) recruiting more diverse samples across demographic and institutional variables to enhance external validity, and (3) incorporating objective performance-based assessments, such as simulation-based evaluations or supervisor ratings, to provide more accurate indicators of decision-making competence.

## 6 Conclusion

This study demonstrates that both cognitive and relational self-directed learning are significant mediating pathways in the association between emotional intelligence and clinical decision-making confidence among registered nurses. Examined within structurally independent models, cognitive self-directed learning accounted for 71.8% of the total standardized effect in its respective model, and relational self-directed learning accounted for 50.8% in a parallel model, with both indirect effects statistically significant. These findings suggest that professional development programs integrating emotional intelligence training with both metacognitive skill development and collaborative learning initiatives may more effectively foster clinical decision-making confidence than approaches targeting either domain in isolation. Longitudinal and quasi-experimental designs are needed to establish causal directionality, and future research would benefit from more diverse nursing samples and objective performance-based assessments.

### 6.1 Glossary

**Clinical Decision-Making Confidence**: A nurse’s self-assurance in their ability to identify patient problems, interpret clinical data, implement appropriate interventions, and evaluate care effectiveness across various clinical situations.

**Cognitive Self-Directed Learning**: The internal cognitive processes of autonomous learning, including learning awareness, learning strategies, learning behavior, and learning evaluation that enable independent knowledge acquisition and skill development.

**Emotional Intelligence**: The multidimensional capacity to recognize, understand, and effectively manage both personal emotions and those of others within professional contexts, encompassing self-emotional appraisal, others’ emotional appraisal, emotional regulation, and emotional utilization.

**Self-Directed Learning**: An individual’s capacity to take initiative in diagnosing learning needs, formulating learning goals, identifying resources, implementing strategies, and evaluating learning outcomes independently within professional contexts.

**Social and Relational Self-Directed Learning**: The interpersonal and collaborative dimensions of autonomous learning that involve building relationships with mentors, peers, and colleagues to support professional development and knowledge acquisition.

### 6.2 Declaration of generative AI and AI-assisted technologies in the writing process

During the preparation of this work, the author(s) used ChatGPT by OpenAI to improve the readability and language of the manuscript. After using this tool, the author(s) carefully reviewed and edited the content as needed and take(s) full responsibility for the content of the published article.

### Supporting information

S1 Checklist STROBE checklist for cross-sectional studies. **Legend:** This checklist demonstrates adherence to the Strengthening the Reporting of Observational Studies in Epidemiology (STROBE) guidelines for reporting cross-sectional studies.
(DOCX)

S2 Table Standardized Factor Loadings, Composite Reliability, and Average Variance Extracted of All Measurement Instruments. **Legend:** This table presents the standardized factor loadings for all items derived from confirmatory factor analysis, along with composite reliability and average variance extracted for each construct.
(DOCX)

S3 Table Post-hoc tests results of univariate analysis. **Legend:** This table shows the results of Bonferroni post-hoc tests for significant variables identified in the univariate analysis between different groups.
(DOCX)

S4 Table Descriptive statistics and correlation analysis of study variables. **Legend:** This table presents the dimensional scores and total scores of each questionnaire, along with correlation analysis results among all study variables including emotional intelligence, self-directed learning, and clinical decision-making confidence.
(DOCX)

## Abbreviations

C-SDL: Cognitive Self-Directed Learning
CDMC: Clinical Decision-Making Confidence
CDMSCS: Clinical Decision-Making Self-Confidence Scale
EI: Emotional Intelligence
SDL: Self-Directed Learning
SR-SDL: Social and Relational Self-Directed Learning
SRSSDL: Self-Rating Scale of Self-Directed Learning
WLEIS: Wong and Law Emotional Intelligence Scale
