# Supplementary material for: Enhancing nurses’ clinical decision-making confidence through dual pathways of self-directed learning: A structural equation model
Source: PLoS One. 2026 Jun 11;21(6):e0351551. doi: 10.1371/journal.pone.0351551 (PMC13257989; doi:10.1371/journal.pone.0351551)
Supplement: S2 Table — Legend: This table presents the standardized factor loadings for all items derived from confirmatory factor analysis, along with composite reliability and average variance extracted for each construct. (DOCX) [file pone.0351551.s002.docx]

**Standardized Factor Loadings, Composite Reliability (CR), and Average Variance Extracted (AVE) of All Measurement Instruments**

| **Scale** | **Dimension** | **Item** | **Standardized Loading** | **CR** | **AVE** |
| --- | --- | --- | --- | --- | --- |
| Wong and Law Emotional Intelligence Scale | Self-Emotion Appraisal | EI1 | 0.577 | 0.761 | 0.443 |
|  |  | EI2 | 0.720 |  |  |
|  |  | EI3 | 0.722 |  |  |
|  |  | EI4 | 0.657 |  |  |
|  | Others-Emotion Appraisal | EI5 | 0.571 | 0.689 | 0.357 |
|  |  | EI6 | 0.632 |  |  |
|  |  | EI7 | 0.566 |  |  |
|  |  | EI8 | 0.622 |  |  |
|  | Use of Emotion | EI9 | 0.613 | 0.720 | 0.391 |
|  |  | EI10 | 0.648 |  |  |
|  |  | EI11 | 0.644 |  |  |
|  |  | EI12 | 0.602 |  |  |
|  | Regulation of Emotion | EI13 | 0.680 | 0.795 | 0.492 |
|  |  | EI14 | 0.787 |  |  |
|  |  | EI15 | 0.768 |  |  |
|  |  | EI16 | 0.594 |  |  |
| Clinical Decision-Making Self-Confidence Scale | Identification | DC1 | 0.684 | 0.658 | 0.395 |
|  |  | DC2 | 0.708 |  |  |
|  |  | DC3 | 0.489 |  |  |
|  | Assessment | DC4 | 0.649 | 0.595 | 0.331 |
|  |  | DC5 | 0.580 |  |  |
|  |  | DC6 | 0.491 |  |  |
|  | Intervention | DC7 | 0.566 | 0.565 | 0.304 |
|  |  | DC8 | 0.535 |  |  |
|  |  | DC9 | 0.548 |  |  |
|  | Evaluation | DC10 | 0.634 | 0.614 | 0.348 |
|  |  | DC11 | 0.562 |  |  |
|  |  | DC12 | 0.571 |  |  |
| Self-Rating Scale of Self-Directed Learning | Learning Awareness | SDL1 | 0.453 | 0.736 | 0.190 |
|  |  | SDL2 | 0.496 |  |  |
|  |  | SDL3 | 0.394 |  |  |
|  |  | SDL4 | 0.458 |  |  |
|  |  | SDL5 | 0.510 |  |  |
|  |  | SDL6 | 0.534 |  |  |
|  |  | SDL7 | 0.548 |  |  |
|  |  | SDL8 | 0.578 |  |  |
|  |  | SDL9 | 0.279 |  |  |
|  |  | SDL10 | 0.347 |  |  |
|  |  | SDL11 | 0.511 |  |  |
|  |  | SDL12 | 0.363 |  |  |
|  | Learning Strategies | SDL13 | 0.368 | 0.780 | 0.233 |
|  |  | SDL14 | 0.349 |  |  |
|  |  | SDL15 | 0.390 |  |  |
|  |  | SDL16 | 0.464 |  |  |
|  |  | SDL17 | 0.563 |  |  |
|  |  | SDL18 | 0.498 |  |  |
|  |  | SDL19 | 0.616 |  |  |
|  |  | SDL20 | 0.538 |  |  |
|  |  | SDL21 | 0.571 |  |  |
|  |  | SDL22 | 0.417 |  |  |
|  |  | SDL23 | 0.497 |  |  |
|  |  | SDL24 | 0.494 |  |  |
|  | Learning Behavior | SDL25 | 0.522 | 0.807 | 0.271 |
|  |  | SDL26 | 0.521 |  |  |
|  |  | SDL27 | 0.514 |  |  |
|  |  | SDL28 | 0.468 |  |  |
|  |  | SDL29 | 0.520 |  |  |
|  |  | SDL30 | 0.546 |  |  |
|  |  | SDL31 | 0.548 |  |  |
|  |  | SDL32 | 0.478 |  |  |
|  |  | SDL33 | 0.584 |  |  |
|  |  | SDL34 | 0.596 |  |  |
|  |  | SDL35 | 0.455 |  |  |
|  |  | SDL36 | 0.313 |  |  |
|  | Learning Evaluation | SDL37 | 0.512 | 0.838 | 0.306 |
|  |  | SDL38 | 0.554 |  |  |
|  |  | SDL39 | 0.575 |  |  |
|  |  | SDL40 | 0.488 |  |  |
|  |  | SDL41 | 0.483 |  |  |
|  |  | SDL42 | 0.542 |  |  |
|  |  | SDL43 | 0.572 |  |  |
|  |  | SDL44 | 0.607 |  |  |
|  |  | SDL45 | 0.592 |  |  |
|  |  | SDL46 | 0.579 |  |  |
|  |  | SDL47 | 0.538 |  |  |
|  |  | SDL48 | 0.545 |  |  |
|  | Interpersonal Relationships | SDL49 | 0.527 | 0.803 | 0.257 |
|  |  | SDL50 | 0.491 |  |  |
|  |  | SDL51 | 0.582 |  |  |
|  |  | SDL52 | 0.535 |  |  |
|  |  | SDL53 | 0.516 |  |  |
|  |  | SDL54 | 0.472 |  |  |
|  |  | SDL55 | 0.486 |  |  |
|  |  | SDL56 | 0.477 |  |  |
|  |  | SDL57 | 0.488 |  |  |
|  |  | SDL58 | 0.534 |  |  |
|  |  | SDL59 | 0.466 |  |  |
|  |  | SDL60 | 0.502 |  |  |
| **Note:** All factor loadings were statistically significant (*p* < 0.01**). While most CR values met acceptable thresholds, several dimensions (particularly within SRSSDL) showed AVE values below the conventional criterion of 0.50, indicating modest convergent validity. Given the large number of items per dimension and adequate CR levels, the constructs were retained to preserve theoretical completeness. | | | | | |
