# Supplementary material for: Enhancing nurses’ clinical decision-making confidence through dual pathways of self-directed learning: A structural equation model
Source: PLoS One. 2026 Jun 11;21(6):e0351551. doi: 10.1371/journal.pone.0351551 (PMC13257989; doi:10.1371/journal.pone.0351551)
Supplement: S3 Table — Legend: This table shows the results of Bonferroni post-hoc tests for significant variables identified in the univariate analysis between different groups. (DOCX) [file pone.0351551.s003.docx]

**Outcome:** Clinical Decision-Making Self-Confidence Scale

1.Marital Status

Method: LSD

| (I) Marital Status | (J) Marital Status | Mean Difference (I-J) | Std. error | *p* value | 95% Confidence interval | |
| --- | --- | --- | --- | --- | --- | --- |
|  |  |  |  |  | Lower bound | Upper bound |
| Single | Married | -2.44 | 0.40 | ＜0.01 ** | -3.23 | -1.65 |
|  | Divorced or Widowed | -2.69 | 1.17 | 0.02* | -4.99 | -0.39 |
| Married | Single | 2.44 | 0.40 | ＜0.01 ** | 1.65 | 3.23 |
|  | Divorced or Widowed | -0.25 | 1.14 | 0.83 | -2.47 | 1.98 |
| Divorced or Widowed | Single | 2.69 | 1.17 | 0.02* | 0.39 | 4.99 |
|  | Married | 0.25 | 1.14 | 0.83 | -1.98 | 2.47 |
| **p* < 0.05; ***p* < 0.01. | | | | | | |

2.Employment Type

Method: LSD

| (I) Employment Type | (J) Employment Type | Mean Difference (I-J) | Std. error | *p* value | 95% Confidence interval | |
| --- | --- | --- | --- | --- | --- | --- |
|  |  |  |  |  | Lower bound | Upper bound |
| Labor Dispatch | Contract | -2.14 | 0.62 | ＜0.01 ** | -3.35 | -0.93 |
|  | Permanent | -1.74 | 0.46 | ＜0.01 ** | -2.65 | -0.84 |
| Contract | Labor Dispatch | 2.14 | 0.62 | ＜0.01 ** | 0.93 | 3.35 |
|  | Permanent | 0.40 | 0.50 | 0.43 | -0.59 | 1.39 |
| Permanent | Labor Dispatch | 1.74 | 0.46 | ＜0.01 ** | 0.84 | 2.65 |
|  | Contract | -0.40 | 0.50 | 0.43 | -1.39 | 0.59 |
| **p* < 0.05; ***p* < 0.01. | | | | | | |

3.Age

Method: LSD

| (I) Age | (J) Age | Mean Difference (I-J) | Std. error | *p* value | 95% Confidence interval | |
| --- | --- | --- | --- | --- | --- | --- |
|  |  |  |  |  | Lower bound | Upper bound |
| 20-30 years | 30-40 years | -2.403 | 0.437 | ＜0.01 ** | -3.26 | -1.55 |
|  | 40-50 years | -3.542 | 0.482 | ＜0.01 ** | -4.49 | -2.6 |
|  | ＞50 years | -3.333 | 0.67 | ＜0.01 ** | -4.65 | -2.02 |
| 30-40 years | 20-30 years | 2.403 | 0.437 | ＜0.01 ** | 1.55 | 3.26 |
|  | 40-50 years | -1.14 | 0.428 | ＜0.01 ** | -1.98 | -0.3 |
|  | ＞50 years | -0.93 | 0.632 | 0.142 | -2.17 | 0.31 |
| 40-50 years | 20-30 years | 3.542 | 0.482 | ＜0.01 ** | 2.6 | 4.49 |
|  | 30-40 years | 1.14 | 0.428 | ＜0.01 ** | 0.3 | 1.98 |
|  | ＞50 years | 0.209 | 0.664 | 0.752 | -1.09 | 1.51 |
| ＞50 years | 20-30 years | 3.333 | 0.67 | ＜0.01 ** | 2.02 | 4.65 |
|  | 30-40 years | 0.93 | 0.632 | 0.142 | -0.31 | 2.17 |
|  | 40-50 years | -0.209 | 0.664 | 0.752 | -1.51 | 1.09 |
| **p* < 0.05; ***p* < 0.01. | | | | | | |

4.Job Title

Method: LSD

| (I) Job Title | (J ) Job Title | Mean Difference (I-J) | Std. error | *p* value | 95% Confidence interval | |
| --- | --- | --- | --- | --- | --- | --- |
|  |  |  |  |  | Lower bound | Upper bound |
| Nurse | Senior Nurse | -2.265 | 0.654 | ＜0.01 ** | -3.55 | -0.98 |
|  | Charge Nurse | -3.428 | 0.63 | ＜0.01 ** | -4.66 | -2.19 |
|  | Deputy Chief or Chief Nurse | -5.523 | 0.794 | ＜0.01 ** | -7.08 | -3.96 |
| Senior Nurse | Nurse | 2.265 | 0.654 | ＜0.01 ** | 0.98 | 3.55 |
|  | Charge Nurse | -1.163 | 0.387 | ＜0.01 ** | -1.92 | -0.4 |
|  | Deputy Chief or Chief Nurse | -3.257 | 0.619 | ＜0.01 ** | -4.47 | -2.04 |
| Charge Nurse | Nurse | 3.428 | 0.63 | ＜0.01 ** | 2.19 | 4.66 |
|  | Senior Nurse | 1.163 | 0.387 | ＜0.01 ** | 0.4 | 1.92 |
|  | Deputy Chief or Chief Nurse | -2.095 | 0.594 | ＜0.01 ** | -3.26 | -0.93 |
| Deputy Chief or Chief Nurse | Nurse | 5.523 | 0.794 | ＜0.01 ** | 3.96 | 7.08 |
|  | Senior Nurse | 3.257 | 0.619 | ＜0.01 ** | 2.04 | 4.47 |
|  | Charge Nurse | 2.095 | 0.594 | ＜0.01 ** | 0.93 | 3.26 |
| **p* < 0.05; ***p* < 0.01. | | | | | | |

5.Years of Work Experience

Method: Turkey HSD

| (I) Years of Work Experience | (J ) Years of Work Experience | Mean Difference (I-J) | Std. error | *p* value | 95% Confidence interval | |
| --- | --- | --- | --- | --- | --- | --- |
|  |  |  |  |  | Lower bound | Upper bound |
| 1-5 years | 6-10 years | -2.713 | 0.577 | ＜0.01 ** | -3.84 | -1.58 |
|  | 11-15 years | -2.942 | 0.518 | ＜0.01 ** | -3.96 | -1.93 |
|  | 16-20 years | -2.675 | 0.58 | ＜0.01 ** | -3.81 | -1.54 |
|  | ＞20 years | -4.867 | 0.534 | ＜0.01 ** | -5.91 | -3.82 |
| 6-10 years | 1-5 years | 2.713 | 0.577 | ＜0.01 ** | 1.58 | 3.84 |
|  | 11-15 years | -0.229 | 0.531 | 0.667 | -1.27 | 0.81 |
|  | 16-20 years | 0.038 | 0.592 | 0.949 | -1.12 | 1.2 |
|  | ＞20 years | -2.154 | 0.547 | ＜0.01 ** | -3.23 | -1.08 |
| 11-15 years | 1-5 years | 2.942 | 0.518 | ＜0.01 ** | 1.93 | 3.96 |
|  | 6-10 years | 0.229 | 0.531 | 0.667 | -0.81 | 1.27 |
|  | 16-20 years | 0.267 | 0.535 | 0.618 | -0.78 | 1.32 |
|  | ＞20 years | -1.925 | 0.485 | ＜0.01 ** | -2.88 | -0.97 |
| 16-20 years | 1-5 years | 2.675 | 0.58 | ＜0.01 ** | 1.54 | 3.81 |
|  | 6-10 years | -0.038 | 0.592 | 0.949 | -1.2 | 1.12 |
|  | 11-15 years | -0.267 | 0.535 | 0.618 | -1.32 | 0.78 |
|  | ＞20 years | -2.192 | 0.551 | ＜0.01 ** | -3.27 | -1.11 |
| ＞20 years | 1-5 years | 4.867 | 0.534 | ＜0.01 ** | 3.82 | 5.91 |
|  | 6-10 years | 2.154 | 0.547 | ＜0.01 ** | 1.08 | 3.23 |
|  | 11-15 years | 1.925 | 0.485 | ＜0.01 ** | 0.97 | 2.88 |
|  | 16-20 years | 2.192 | 0.551 | ＜0.01 ** | 1.11 | 3.27 |
| **p* < 0.05; ***p* < 0.01. | | | | | | |

6.Department

Method: Turkey HSD

| (I) Department | (J ) Department | Mean Difference (I-J) | Std. error | *p* value | 95% Confidence interval | |
| --- | --- | --- | --- | --- | --- | --- |
|  |  |  |  |  | Lower bound | Upper bound |
| Internal Medicine | Surgery | 0.555 | 0.447 | 0.215 | -0.32 | 1.43 |
|  | ICU | -0.369 | 0.903 | 0.683 | -2.14 | 1.4 |
|  | Emergency | 1.342 | 0.953 | 0.159 | -0.53 | 3.21 |
|  | Operating Room | 1.366 | 0.677 | 0.044 | 0.04 | 2.69 |
|  | Pediatrics | 1.89 | 1.338 | 0.158 | -0.73 | 4.51 |
|  | Gynecology | -0.264 | 1.338 | 0.844 | -2.89 | 2.36 |
|  | Obstetrics | 4.152 | 0.878 | ＜0.01 ** | 2.43 | 5.87 |
|  | Other | 0.31 | 0.528 | 0.557 | -0.73 | 1.35 |
| Surgery | Internal Medicine | -0.555 | 0.447 | 0.215 | -1.43 | 0.32 |
|  | ICU | -0.924 | 0.913 | 0.312 | -2.71 | 0.87 |
|  | Emergency | 0.786 | 0.963 | 0.414 | -1.1 | 2.67 |
|  | Operating Room | 0.81 | 0.691 | 0.241 | -0.54 | 2.17 |
|  | Pediatrics | 1.335 | 1.344 | 0.321 | -1.3 | 3.97 |
|  | Gynecology | -0.819 | 1.344 | 0.542 | -3.46 | 1.82 |
|  | Obstetrics | 3.596 | 0.888 | ＜0.01 ** | 1.85 | 5.34 |
|  | Other | -0.245 | 0.545 | 0.653 | -1.31 | 0.82 |
| ICU | Internal Medicine | 0.369 | 0.903 | 0.683 | -1.4 | 2.14 |
|  | Surgery | 0.924 | 0.913 | 0.312 | -0.87 | 2.71 |
|  | Emergency | 1.71 | 1.242 | 0.169 | -0.73 | 4.15 |
|  | Operating Room | 1.734 | 1.045 | 0.097 | -0.32 | 3.78 |
|  | Pediatrics | 2.259 | 1.556 | 0.147 | -0.79 | 5.31 |
|  | Gynecology | 0.105 | 1.556 | 0.946 | -2.95 | 3.16 |
|  | Obstetrics | 4.52 | 1.185 | ＜0.01 ** | 2.2 | 6.84 |
|  | Other | 0.678 | 0.955 | 0.477 | -1.19 | 2.55 |
| Emergency | Internal Medicine | -1.342 | 0.953 | 0.159 | -3.21 | 0.53 |
|  | Surgery | -0.786 | 0.963 | 0.414 | -2.67 | 1.1 |
|  | ICU | -1.71 | 1.242 | 0.169 | -4.15 | 0.73 |
|  | Operating Room | 0.024 | 1.089 | 0.982 | -2.11 | 2.16 |
|  | Pediatrics | 0.548 | 1.586 | 0.73 | -2.56 | 3.66 |
|  | Gynecology | -1.605 | 1.586 | 0.312 | -4.72 | 1.51 |
|  | Obstetrics | 2.81 | 1.223 | 0.02* | 0.41 | 5.21 |
|  | Other | -1.032 | 1.003 | 0.304 | -3 | 0.94 |
| Operating Room | Internal Medicine | -1.366 | 0.677 | 0.044 | -2.69 | -0.04 |
|  | Surgery | -0.81 | 0.691 | 0.241 | -2.17 | 0.54 |
|  | ICU | -1.734 | 1.045 | 0.097 | -3.78 | 0.32 |
|  | Emergency | -0.024 | 1.089 | 0.982 | -2.16 | 2.11 |
|  | Pediatrics | 0.524 | 1.437 | 0.715 | -2.3 | 3.34 |
|  | Gynecology | -1.629 | 1.437 | 0.257 | -4.45 | 1.19 |
|  | Obstetrics | 2.786 | 1.023 | ＜0.01 ** | 0.78 | 4.79 |
|  | Other | -1.056 | 0.746 | 0.157 | -2.52 | 0.41 |
| Pediatrics | Internal Medicine | -1.89 | 1.338 | 0.158 | -4.51 | 0.73 |
|  | Surgery | -1.335 | 1.344 | 0.321 | -3.97 | 1.3 |
|  | ICU | -2.259 | 1.556 | 0.147 | -5.31 | 0.79 |
|  | Emergency | -0.548 | 1.586 | 0.73 | -3.66 | 2.56 |
|  | Operating Room | -0.524 | 1.437 | 0.715 | -3.34 | 2.3 |
|  | Gynecology | -2.154 | 1.843 | 0.243 | -5.77 | 1.46 |
|  | Obstetrics | 2.262 | 1.542 | 0.143 | -0.76 | 5.29 |
|  | Other | -1.58 | 1.373 | 0.25 | -4.27 | 1.11 |
| Gynecology | Internal Medicine | 0.264 | 1.338 | 0.844 | -2.36 | 2.89 |
|  | Surgery | 0.819 | 1.344 | 0.542 | -1.82 | 3.46 |
|  | ICU | -0.105 | 1.556 | 0.946 | -3.16 | 2.95 |
|  | Emergency | 1.605 | 1.586 | 0.312 | -1.51 | 4.72 |
|  | Operating Room | 1.629 | 1.437 | 0.257 | -1.19 | 4.45 |
|  | Pediatrics | 2.154 | 1.843 | 0.243 | -1.46 | 5.77 |
|  | Obstetrics | 4.415 | 1.542 | ＜0.01 ** | 1.39 | 7.44 |
|  | Other | 0.574 | 1.373 | 0.676 | -2.12 | 3.27 |
| Obstetrics | Internal Medicine | -4.152 | 0.878 | ＜0.01 ** | -5.87 | -2.43 |
|  | Surgery | -3.596 | 0.888 | ＜0.01 ** | -5.34 | -1.85 |
|  | ICU | -4.52 | 1.185 | ＜0.01 ** | -6.84 | -2.2 |
|  | Emergency | -2.81 | 1.223 | 0.02* | -5.21 | -0.41 |
|  | Operating Room | -2.786 | 1.023 | ＜0.01 ** | -4.79 | -0.78 |
|  | Pediatrics | -2.262 | 1.542 | 0.143 | -5.29 | 0.76 |
|  | Gynecology | -4.415 | 1.542 | ＜0.01 ** | -7.44 | -1.39 |
|  | Other | -3.842 | 0.931 | ＜0.01 ** | -5.67 | -2.01 |
| Other | Internal Medicine | -0.31 | 0.528 | 0.557 | -1.35 | 0.73 |
|  | Surgery | 0.245 | 0.545 | 0.653 | -0.82 | 1.31 |
|  | ICU | -0.678 | 0.955 | 0.477 | -2.55 | 1.19 |
|  | Emergency | 1.032 | 1.003 | 0.304 | -0.94 | 3 |
|  | Operating Room | 1.056 | 0.746 | 0.157 | -0.41 | 2.52 |
|  | Pediatrics | 1.58 | 1.373 | 0.25 | -1.11 | 4.27 |
|  | Gynecology | -0.574 | 1.373 | 0.676 | -3.27 | 2.12 |
|  | Obstetrics | 3.842 | 0.931 | ＜0.01 ** | 2.01 | 5.67 |
| **p* < 0.05; ***p* < 0.01. | | | | | | |
