# Supplementary material for: Enhancing nurses’ clinical decision-making confidence through dual pathways of self-directed learning: A structural equation model
Source: PLoS One. 2026 Jun 11;21(6):e0351551. doi: 10.1371/journal.pone.0351551 (PMC13257989; doi:10.1371/journal.pone.0351551)
Supplement: S4 Table — Legend: This table presents the dimensional scores and total scores of each questionnaire, along with correlation analysis results among all study variables including emotional intelligence, self-directed learning, and clinical decision-making confidence. (DOCX) [file pone.0351551.s004.docx]

**Descriptive analysis (n = 1478)**

| **Item** | **Total score ranges** | **Total scores (M ± SD)** | **Average scores (M ± SD)** |
| --- | --- | --- | --- |
| **Wong and Law Emotional Intelligence Scale** |  |  |  |
| Self-Emotion Appraisal | 10-28 | 24.49±3.49 | 6.12±0.87 |
| Others-Emotion Appraisal | 8-28 | 21.89±3.70 | 5.47±0.93 |
| Use of Emotion | 4-28 | 21.92±3.92 | 5.48±0.98 |
| Regulation of Emotion | 7-28 | 20.63±4.49 | 5.16±1.12 |
| **Clinical Decision-Making Self-Confidence Scale** |  |  |  |
| Identification | 6-15 | 12.23±1.71 | 4.08±0.57 |
| Assessment | 6-15 | 11.77±1.58 | 3.92±0.52 |
| Intervention | 6-15 | 11.91±1.59 | 3.97±0.53 |
| Evaluation | 7-15 | 11.69±1.60 | 3.90±0.53 |
| **Self-Rating Scale of Self-Directed Learning** |  |  |  |
| Learning Awareness | 34-60 | 48.14±4.96 | 4.01±0.41 |
| Learning Strategies | 30-60 | 47.59±5.63 | 3.97±0.47 |
| Learning Behavior | 28-60 | 45.72±6.40 | 3.81±0.53 |
| Learning Evaluation | 23-60 | 47.72±6.16 | 3.98±0.51 |
| Interpersonal Relationships | 33-60 | 48.04±6.00 | 4.00±0.50 |
| **Note:** The Wong and Law Emotional Intelligence Scale uses a 7-point Likert scale, whereas the Clinical Decision-Making Self-Confidence Scale and the Self-Rating Scale of Self-Directed Learning use 5-point Likert scales. Total score ranges reflect the observed minimum and maximum in this sample. M = mean; SD = standard deviation. Higher scores indicate higher levels of the corresponding construct. | | | |

**Correlations analysis (n = 1478)**

| **Pearson correlation analysis** | **1** | **2** | **3** | **4** | **5** | **6** | **7** | **8** | **9** | **10** | **11** | **12** | **13** | **14** | **15** | **16** |
| --- | --- | --- | --- | --- | --- | --- | --- | --- | --- | --- | --- | --- | --- | --- | --- | --- |
| 1.Self-Emotion Appraisal | 1 | 0.454** | 0.562** | 0.475** | 0.764** | 0.292** | 0.290** | 0.275** | 0.274** | 0.318** | 0.407** | 0.378** | 0.295** | 0.324** | 0.364** | 0.393** |
| 2.Others-Emotion Appraisal | 0.454** | 1 | 0.515** | 0.403** | 0.733** | 0.215** | 0.250** | 0.221** | 0.227** | 0.257** | 0.338** | 0.325** | 0.331** | 0.328** | 0.397** | 0.386** |
| 3.Use of Emotion | 0.562** | 0.515** | 1 | 0.652** | 0.861** | 0.374** | 0.377** | 0.344** | 0.351** | 0.407** | 0.533** | 0.508** | 0.472** | 0.496** | 0.525** | 0.567** |
| 4.Regulation of Emotion | 0.475** | 0.403** | 0.652** | 1 | 0.820** | 0.319** | 0.306** | 0.297** | 0.320** | 0.350** | 0.423** | 0.390** | 0.391** | 0.397** | 0.441** | 0.458** |
| 5.Wong and Law Emotional Intelligence Scale | 0.764** | 0.733** | 0.861** | 0.820** | 1 | 0.379** | 0.385** | 0.358** | 0.371** | 0.420** | 0.535** | 0.504** | 0.471** | 0.488** | 0.544** | 0.569** |
| 6.Identification | 0.292** | 0.215** | 0.374** | 0.319** | 0.379** | 1 | 0.710** | 0.656** | 0.626** | 0.847** | 0.465** | 0.440** | 0.432** | 0.395** | 0.432** | 0.484** |
| 7.Assessment | 0.290** | 0.250** | 0.377** | 0.306** | 0.385** | 0.710** | 1 | 0.775** | 0.746** | 0.906** | 0.458** | 0.438** | 0.472** | 0.423** | 0.448** | 0.502** |
| 8.Intervention | 0.275** | 0.221** | 0.344** | 0.297** | 0.358** | 0.656** | 0.775** | 1 | 0.816** | 0.910** | 0.479** | 0.463** | 0.489** | 0.442** | 0.480** | 0.528** |
| 9.Evaluation | 0.274** | 0.227** | 0.351** | 0.320** | 0.371** | 0.626** | 0.746** | 0.816** | 1 | 0.894** | 0.493** | 0.486** | 0.486** | 0.453** | 0.494** | 0.541** |
| 10.Clinical Decision-Making Self-Confidence Scale | 0.318** | 0.257** | 0.407** | 0.350** | 0.420** | 0.847** | 0.906** | 0.910** | 0.894** | 1 | 0.533** | 0.514** | 0.528** | 0.481** | 0.521** | 0.578** |
| 11.Learning Awareness | 0.407** | 0.338** | 0.533** | 0.423** | 0.535** | 0.465** | 0.458** | 0.479** | 0.493** | 0.533** | 1 | 0.723** | 0.655** | 0.684** | 0.679** | 0.828** |
| 12.Learning Strategies | 0.378** | 0.325** | 0.508** | 0.390** | 0.504** | 0.440** | 0.438** | 0.463** | 0.486** | 0.514** | 0.723** | 1 | 0.743** | 0.739** | 0.735** | 0.883** |
| 13.Learning Behavior | 0.295** | 0.331** | 0.472** | 0.391** | 0.471** | 0.432** | 0.472** | 0.489** | 0.486** | 0.528** | 0.655** | 0.743** | 1 | 0.821** | 0.768** | 0.905** |
| 14.Learning Evaluation | 0.324** | 0.328** | 0.496** | 0.397** | 0.488** | 0.395** | 0.423** | 0.442** | 0.453** | 0.481** | 0.684** | 0.739** | 0.821** | 1 | 0.825** | 0.921** |
| 15.Interpersonal Relationships | 0.364** | 0.397** | 0.525** | 0.441** | 0.544** | 0.432** | 0.448** | 0.480** | 0.494** | 0.521** | 0.679** | 0.735** | 0.768** | 0.825** | 1 | 0.905** |
| 16.Self-Rating Scale of Self-Directed Learning | 0.393** | 0.386** | 0.567** | 0.458** | 0.569** | 0.484** | 0.502** | 0.528** | 0.541** | 0.578** | 0.828** | 0.883** | 0.905** | 0.921** | 0.905** | 1 |
| **p* < 0.05; ***p* < 0.01. | | | | | | | | | | | | | | | | |
