# Supplementary material for: Enhancing nurses’ clinical decision-making confidence through dual pathways of self-directed learning: A structural equation model
Source: PLoS One. 2026 Jun 11;21(6):e0351551. doi: 10.1371/journal.pone.0351551 (PMC13257989; doi:10.1371/journal.pone.0351551)
Supplement: Attachment — Submitted filename: Dual Pathways of Self-Directed Learning Review Report.docx [file pone.0351551.s005.docx]

**Dear Editor and Authors,**

Thank you for the opportunity to review the manuscript entitled “Enhancing Nurses' Clinical Decision-Making Confidence Through Dual Pathways of Self-Directed Learning: A Structural Equation Model.” This is a timely and methodologically robust study that addresses a critical issue in nursing competency and professional development. The application of structural equation modeling to test a dual-mediation hypothesis is a particular strength, and the findings have meaningful implications for nursing education and practice. However, several conceptual and methodological aspects require clarification and refinement to strengthen the manuscript before publication.

-The abstract provides a clear summary of the study's background, methods, and key findings. The reporting of the specific mediation percentages (33.9% for cognitive and 24.1% for relational pathways) is a strength. However, the abstract would be enhanced by stating the potential score range for the Clinical Decision-Making Self-Confidence Scale (mean = 47.03±6.69) to give readers proper context for interpreting "low to moderate" confidence levels.

-The introduction effectively establishes the demands of modern healthcare systems and provides solid definitions for the core constructs (EI, SDL, CDMC). The theoretical rationale, drawing on Self-Determination Theory and Experiential Learning Theory, is well-articulated and provides a strong foundation for the hypotheses. This section would benefit from a clearer initial statement of the scoping review's primary aim and its contribution to addressing the identified research gap.

-The methods section is generally well-described, with a large sample size and the use of validated instruments. The adherence to ethical guidelines is clear. However, the sample is overwhelmingly female (97.56%) and from tertiary hospitals, which is a significant limitation for the generalizability of the findings and should be more prominently acknowledged as a core constraint of the study, not just a future research suggestion.

-The most critical issue pertains to the conceptualization and measurement of the "Social and Relational Self-Directed Learning (SR-SDL)" construct. The manuscript states this is a reconceptualization based on Garrison's model, yet it is operationalized using a single dimension: "Interpersonal Relationships." This is a severe oversimplification of a theoretically rich construct. A "social and relational" dimension should conceptually encompass collaboration, peer learning, mentorship, and feedback-seeking. Using one subscale threatens the construct's validity and weakens the interpretation of the path analysis. The authors must either re-label the construct more accurately (e.g., "Interpersonal Relationship Skills in Learning") or provide a more robust psychometric justification for the two-factor model.

-The results are comprehensively presented with appropriate statistical support. A major inconsistency must be resolved: **Table 1** presents demographic data for an n of 1478, while the text consistently states the analytical sample is 1024. This discrepancy is confusing and undermines the clarity of the results. The table must be corrected to reflect the final analytical sample. Furthermore, the path coefficient from EI to Relational SDL is reported as β = 1.093, which is unusually high for a standardized coefficient. This should be verified and reported correctly.

-The discussion effectively interprets the findings, particularly the dominance of the cognitive pathway. The link back to Self-Determination Theory is well-executed.

-The finding of a complete absence of family/carer involvement in the reviewed studies is a striking indicator of a gap in person-centered care within prison health and deserves to be a central point of the discussion. The institutional and security-related barriers that likely cause this omission should be discussed, emphasizing why this represents a critical failure of person-centered care in prisons.

-The most striking finding, the complete absence of measures related to family and carer involvement, deserves to be elevated to a central point of the discussion, perhaps in its own paragraph. The institutional and security-related barriers that likely cause this gap should be discussed, emphasizing why its absence represents a critical failure of person-centred care in prisons.

-Please confirm and standardize the count of survey-based studies throughout the manuscript. The abstract states 21 studies (52%) utilized surveys, while the body mentions 24 studies. The correct number must be uniformly applied.

-The discussion on power imbalances and lack of autonomy is excellent. To strengthen the theoretical framing, these issues should be more explicitly linked to established concepts like "structural stigma" or "coercive environments" that are intrinsic to the carceral setting and fundamentally shape patient-provider interactions.

-The call for new tools is clear and would be strengthened by explicitly recommending their co-design with people who have lived experience of incarceration. This aligns with participatory research paradigms and is crucial for ensuring the resulting tools are contextually valid and meaningful.

-While limitations are acknowledged, the discussion could more directly address how the focus on explicit patient experience measures in titles/abstracts may have omitted relevant data embedded in broader studies of health in prisons.

-The conclusion succinctly summarizes the review's contributions. The final sentence could be more powerful by directly echoing the primary recommendation, for example: "...to address health inequities faced by people experiencing incarceration through the co-design, validation, and implementation of context-specific patient experience measures."

-The statement on page 35 that self-directed learning has a "58% total mediation rate" (33.9% + 24.1%) is a statistical misinterpretation. The two mediators are correlated components of the same broader construct (SDL), and their effects are not additive. The discussion should focus on comparing the two pathways rather than summing their effects.

-The cross-sectional design is noted as a limitation, but the language in the results and discussion often implies causality (e.g., "emotional intelligence positively influenced..."). The manuscript must consistently use correlational language (e.g., "was associated with," "predicted") to avoid causal claims.

-There is a significant chronological inconsistency: the manuscript states data was collected "between June and August 2025," which is in the future. Furthermore, several references are from 2024 and 2025. This must be corrected to reflect the actual data collection timeline, or the status of the 2025 references must be clarified (e.g., "Advance online publication").

A number of recent, contextually relevant studies support the core themes of this manuscript and can be used to strengthen the scholarly grounding.

-In the **Introduction** and **Discussion**, the work of **Aboalrob et al. (2025)**, "Understanding the influence of self-concept on clinical decision-making among nurses" (PLOS ONE), provides direct, independent evidence for studying psychological predictors of CDMC, helping to position your focus on EI within a broader nomological network.

-The study by **Ayed et al. (2025)**, "Insights into the relationship between emotional intelligence and critical thinking among nursing students" (BMC Nursing), offers robust external validation for your Hypothesis 2 (EI -> SDL), as critical thinking is a core component of cognitive self-directed learning. This should be cited when developing your rationale or interpreting this finding.

-The findings of **Batran et al. (2025),** "The relationship between professional quality of life and work environment among nurses..." (PLOS ONE), help contextualize your "relational SDL" pathway by illustrating how the professional environment and interpersonal dynamics are critically linked to nurse outcomes. This supports the discussion of how relational factors impact confidence.

-The paper by **Aqtam et al. (2025)**, "Work engagement and its association with emotional intelligence..." (PLOS ONE), reinforces the significance of EI as a valuable construct in the nursing profession, linking it to positive work-related states and supporting the overall focus of your study.

These references provide clear and strong support for the theoretical framing, methodological considerations, and practical implications of the study and should be incorporated to enhance the manuscript's scholarly impact.

**Suggested reference list**

1. Aboalrob, W., Ayed, A., Malak, M. Z., & Aqtam, I. (2025). Understanding the influence of self-concept on clinical decision-making among nurses: A cross-sectional study. PLOS ONE, 20(8), e0330905. <https://doi.org/10.1371/journal.pone.0330905>
2. Ayed, A., Aqtam, I., Malak, M. Z., et al. (2025). Insights into the relationship between emotional intelligence and critical thinking among nursing students. BMC Nursing, 24, 1107. <https://doi.org/10.1186/s12912-025-03782-7>
3. Batran, A., Aqtam, I., Ayed, A., & Ejheisheh, M. A. (2025). The relationship between professional quality of life and work environment among nurses in neonate care units. PLOS ONE, 20(4), e0322023. <https://doi.org/10.1371/journal.pone.0322023>
4. Aqtam I, Ayed A, Batran A, Ejheisheh MA, Melhem RH, Shouli M (2025) Work engagement and its association with emotional intelligence and demographic characteristics among nurses in Palestinian neonatal intensive care units. PLoS One 20(9): e0332908. <https://doi.org/10.1371/journal.pone.0332908>
