# Supplementary material for: Enhancing nurses’ clinical decision-making confidence through dual pathways of self-directed learning: A structural equation model
Source: PLoS One. 2026 Jun 11;21(6):e0351551. doi: 10.1371/journal.pone.0351551 (PMC13257989; doi:10.1371/journal.pone.0351551)
Supplement: Attachment — Submitted filename: OK-Point by Point Response to Reviewers.docx [file pone.0351551.s007.docx]

**Dear Reviewer #3:**

Thank you for your thoughtful and constructive feedback on our manuscript. We sincerely appreciate your recognition of our study's contribution to the field and your positive evaluation of our theoretical foundation, methodological rigor, and practical implications. Your insightful suggestions have been invaluable in helping us improve the quality and clarity of our work. We are grateful for the time and effort you devoted to reviewing our manuscript, and we believe your recommendations have substantially strengthened the final version. We have carefully considered all your comments and revised our manuscript accordingly. *Revised portions are marked in red in Revised Manuscript with Track Changes.* Below are our point-by-point responses to your comments:

**Comment 1:** To further strengthen the theoretical grounding of the study, the authors may consider engaging with Abdallah and Abdallah (2023), which examines self-directed learning as a mechanism for professional competence development in practice-based disciplines. This work could enrich the discussion of how cognitive and relational self-directed learning pathways contribute to confidence and applied decision-making.

**Response to Comment 1:** We thank the reviewer for this valuable recommendation. Abdallah and Abdallah (2023) offers a well-grounded treatment of self-directed learning as a mechanism for professional competence development in practice-based disciplines, and we agree that engaging with this work meaningfully enriches the discussion of the cognitive self-directed learning pathway in Section 4.2. Accordingly, we have revised the relevant sentence in that paragraph to incorporate the conceptual framing advanced in this study, integrating the reference in a way that deepens the theoretical interpretation of how emotionally intelligent nurses progressively consolidate clinical competence through systematic self-directed learning. We believe this revision strengthens the theoretical coherence of Section 4.2 and is consistent with the reviewer's intent.

**Comment 2:** The Discussion section could be slightly streamlined to reduce repetition, particularly where findings are restated multiple times across subsections.

**Response to Comment 2:** We thank the reviewer for this observation and agree that certain findings were restated with unnecessary redundancy across subsections of the Discussion. In response, we have revised Sections 4.1, 4.2, and 4.3 with the goal of eliminating iterative restatement while preserving the interpretive substance of each argument.

Within Section 4.1, the three subsections addressing the self-directed learning dimensions (4.1.1), emotional utilization (4.1.2), and experience- and department-based predictors (4.1.3) have each been condensed. Specifically, the original four-paragraph treatment of the self-directed learning dimensions in Section 4.1.1 has been consolidated into a single, integrated paragraph that preserves the core interpretive point for each predictor without restating the regression findings already reported in the Results. Sections 4.1.2 and 4.1.3 were similarly tightened by removing hedged restatements of numerical findings and redundant elaboration of points already established. A new subsection, 4.1.4, has also been introduced in response to Reviewer 4 Comment 3 and Reviewer 6 Comment 4, addressing the potential influence of sample homogeneity on the observed confidence patterns. The parallel streamlining applied to Sections 4.2 and 4.3 reduces iterative reference to mediation proportions and effect sizes across paragraphs. These revisions collectively improve the logical coherence and readability of the Discussion without compromising analytical depth.

**Comment 3:** Some interpretations (e.g., department-based differences and experiential explanations) could be presented more concisely to strengthen focus.

**Response to Comment 3:** We appreciate the reviewer's constructive suggestion and agree that the interpretations of department-based differences and work experience in Section 4.1.3 would benefit from greater conciseness. Accordingly, we have condensed both discussions into single, focused sentences that preserve the core interpretive argument while avoiding speculative elaboration. We have also attended carefully to wording throughout this subsection to avoid causal inference, given that cross-sectional data permit the identification of associations rather than directional effects. We believe these revisions sharpen the analytical focus of Section 4.1.3 without sacrificing substantive content.

**Comment 4:** While the conclusions are well supported, briefly acknowledging the cross-sectional design when discussing implications would further strengthen methodological transparency.

**Response to Comment 4:** We appreciate the reviewer's constructive feedback. In response, we have added a brief statement in Section 4.3 (Practical Implications) explicitly acknowledging the cross-sectional design and noting that longitudinal research is needed to confirm causal directionality before the proposed intervention models are broadly implemented. We trust this revision adequately addresses the reviewer's concern regarding methodological transparency.

**Dear Reviewer #4:**

Thank you for your helpful suggestions on our manuscript. We have carefully considered all your comments and revised our manuscript accordingly. *Revised portion are marked in red in Revised Manuscript with Track Changes.* Below are our point-by-point responses to your comments:

**Comment 1:** The abstract provides a clear summary of the study's background, methods, and key findings. The reporting of the specific mediation percentages (33.9% for cognitive and 24.1% for relational pathways) is a strength. However, the abstract would be enhanced by stating the potential score range for the Clinical Decision-Making Self-Confidence Scale (mean = 47.03±6.69) to give readers proper context for interpreting "low to moderate" confidence levels.

**Response to Comment 1:** We appreciate the reviewer's positive assessment of the recognition of the mediation percentages as a strength of the reporting. We must, however, note that those figures required correction following an error identified during the revision process. The original percentages of 33.9% and 24.1% arose from an inadvertent mixing of standardized and unstandardized outputs in the Amos bootstrap analysis, whereby the total effect row in Table 3 had been populated with unstandardized values while all path coefficients were drawn from the standardized solution. Upon correcting the total effect to its standardized estimate, the mediation proportions have been updated to 71.8% for the cognitive self-directed learning pathway and 50.8% for the relational self-directed learning pathway, and all relevant descriptions have been revised accordingly.

Regarding the suggestion to provide score range context for the Clinical Decision-Making Self-Confidence Scale, we thank the reviewer for this constructive recommendation. In addition, another reviewer raised the methodological point that scale scores should be expressed as item averages by dividing the total score by the number of items, so that reported values fall within the original Likert scale range and allow direct interpretation of magnitude. Taking both concerns together, we revised Section 3.2 to report item-average scores for all three instruments, with the potential score range for each scale explicitly stated. The CDMSCS is now reported as an item mean on a 5-point scale, and the characterization of confidence levels has been updated to reflect the revised scoring approach. S4 Table has been comprehensively updated to incorporate these changes, and all relevant descriptions throughout the manuscript have been updated consistently.

**Descriptive analysis (n = 1478)**

| **Item** | **Total score ranges** | **Total scores (M ± SD)** | **Average scores (M ± SD)** |
| --- | --- | --- | --- |
| **Wong and Law Emotional Intelligence Scale** |  |  |  |
| Self-Emotion Appraisal | 10-28 | 24.49±3.49 | 6.12±0.87 |
| Others-Emotion Appraisal | 8-28 | 21.89±3.70 | 5.47±0.93 |
| Use of Emotion | 4-28 | 21.92±3.92 | 5.48±0.98 |
| Regulation of Emotion | 7-28 | 20.63±4.49 | 5.16±1.12 |
| **Clinical Decision-Making Self-Confidence Scale** |  |  |  |
| Identification | 6-15 | 12.23±1.71 | 4.08±0.57 |
| Assessment | 6-15 | 11.77±1.58 | 3.92±0.52 |
| Intervention | 6-15 | 11.91±1.59 | 3.97±0.53 |
| Evaluation | 7-15 | 11.69±1.60 | 3.90±0.53 |
| **Self-Rating Scale of Self-Directed Learning** |  |  |  |
| Learning Awareness | 34-60 | 48.14±4.96 | 4.01±0.41 |
| Learning Strategies | 30-60 | 47.59±5.63 | 3.97±0.47 |
| Learning Behavior | 28-60 | 45.72±6.40 | 3.81±0.53 |
| Learning Evaluation | 23-60 | 47.72±6.16 | 3.98±0.51 |
| Interpersonal Relationships | 33-60 | 48.04±6.00 | 4.00±0.50 |
| **Note:** The Wong and Law Emotional Intelligence Scale uses a 7-point Likert scale, whereas the Clinical Decision-Making Self-Confidence Scale and the Self-Rating Scale of Self-Directed Learning use 5-point Likert scales. Total score ranges reflect the observed minimum and maximum in this sample. M = mean; SD = standard deviation. Higher scores indicate higher levels of the corresponding construct. | | | |

**Comment 2:** The introduction effectively establishes the demands of modern healthcare systems and provides solid definitions for the core constructs (EI, SDL, CDMC). The theoretical rationale, drawing on Self-Determination Theory and Experiential Learning Theory, is well-articulated and provides a strong foundation for the hypotheses. This section would benefit from a clearer initial statement of the scoping review's primary aim and its contribution to addressing the identified research gap.

**Response to Comment 2:** We appreciate the reviewer's positive assessment of the Introduction and agree that a clearer initial statement of the study's primary aim and its specific contribution to addressing the identified research gap would strengthen the opening of this section. In response, we have revised the opening of the Introduction to include a direct statement of the study's primary aim before the theoretical rationale is developed, so that readers are oriented to the study's scope and contribution from the outset. We have also revised the second paragraph to conclude with an explicit identification of the research gap: specifically, that existing scholarship has predominantly examined self-directed learning as a unified construct, leaving the differential mediating roles of its cognitive and relational dimensions in the development of clinical decision-making confidence insufficiently explored. This sequencing — aim first, gap second — ensures that the study's contribution is legible at the point where the literature review is introduced, rather than emerging only after the theoretical framework has been presented. To further tighten the logical architecture of the Introduction, the revised paragraph now concludes with a transitional statement that explicitly connects the identified gap to the study's investigative objectives, establishing a more direct link between the gap description and the formal presentation of Objectives 2 and 3, which examine cognitive and relational self-directed learning as analytically distinct mediating pathways.

**Comment 3:** The methods section is generally well-described, with a large sample size and the use of validated instruments. The adherence to ethical guidelines is clear. However, the sample is overwhelmingly female (97.56%) and from tertiary hospitals, which is a significant limitation for the generalizability of the findings and should be more prominently acknowledged as a core constraint of the study, not just a future research suggestion.

**Response to Comment 3:** We are grateful to the reviewer for this important observation and fully agree that the demographic homogeneity of the sample constitutes a core interpretive constraint rather than a peripheral note confined to the Limitations section. In response, we have added Section 4.1.4 (Impact of Sample Homogeneity on Confidence Patterns) to the Discussion. This section concisely addresses how the tertiary hospital concentration (97.70%) may have contributed to the elevated overall confidence scores and the significant department-based variation observed, and how the predominance of female nurses (97.56%) may have amplified the predictive role of emotional utilization in the regression model. These points are now presented as substantive interpretive qualifications embedded within the Discussion, rather than deferred solely to future research suggestions.

**Comment 4:** The most critical issue pertains to the conceptualization and measurement of the "Social and Relational Self-Directed Learning (SR-SDL)" construct. The manuscript states this is a reconceptualization based on Garrison's model, yet it is operationalized using a single dimension: "Interpersonal Relationships." This is a severe oversimplification of a theoretically rich construct. A "social and relational" dimension should conceptually encompass collaboration, peer learning, mentorship, and feedback-seeking. Using one subscale threatens the construct's validity and weakens the interpretation of the path analysis. The authors must either re-label the construct more accurately (e.g., "Interpersonal Relationship Skills in Learning") or provide a more robust psychometric justification for the two-factor model.

**Response to Comment 4:** We sincerely thank the reviewer for raising this concern, which touches on a genuinely important question of construct validity that we recognize deserves careful consideration.

We would like to respectfully note that the issues identified in this comment substantially overlap with concerns raised by Reviewer 5 (Comment 6) and Reviewer 6 (Comment 3), both of which we have addressed through revisions to Section 2.4.3. In those responses, we restructured the description of the SRSSDL, clarified the item composition of both subscales, and — critically — conducted and reported a comparative confirmatory factor analysis between the two-factor and five-factor solutions to provide empirical grounding for the dimensional reorganization.

Regarding the reviewer's specific concern, we would like to clarify that the conceptual distinction between cognitive and relational learning pathways drew initial inspiration from Yue et al. (2025), but the decision to operationalize SR-SDL via the Interpersonal Relationships subscale was ultimately evaluated and retained on the basis of the present study's own psychometric evidence. As reported in the revised Section 2.4.3, the two-factor model demonstrated acceptable reliability (Cronbach's α = 0.937) and model fit (χ²/df = 2.709, CFI = 0.803, TLI = 0.796, RMSEA = 0.041, SRMR = 0.043), with absolute fit indices falling within conventional thresholds. The comparative CFA further confirmed that, while the five-factor solution yields incrementally better fit on some indices, the two-factor structure is psychometrically defensible and sufficient to support the proposed mediation analyses. In light of this evidence, we have opted to retain the SR-SDL label and operationalization rather than adopt a renamed construct, as the reviewer's alternative suggestion and the psychometric justification route represent equivalent resolutions to the concern raised; the latter, we believe, better preserves theoretical coherence with the study's hypotheses concerning dual learning pathways. We have also made explicit in the revised manuscript that "Interpersonal Relationships" serves as the operational indicator for SR-SDL, so that the scope and boundaries of the measurement are transparent to the reader.

We recognize that the Interpersonal Relationships subscale represents a parsimonious rather than exhaustive operationalization of the broader relational learning construct, and we have acknowledged this as a limitation. We hope that, taken together with the revisions made in response to Reviewer 5 Comment 6 and Reviewer 6 Comment 3, this psychometric justification addresses the reviewer's concern, and we remain grateful for the opportunity to clarify these decisions.

**Comment 5:** The results are comprehensively presented with appropriate statistical support. A major inconsistency must be resolved: Table 1 presents demographic data for an n of 1478, while the text consistently states the analytical sample is 1024. This discrepancy is confusing and undermines the clarity of the results. The table must be corrected to reflect the final analytical sample.

**Response to Comment 5:** We sincerely thank the reviewer for identifying this inconsistency, which we recognize as a significant source of confusion. Upon carefully reviewing the dataset and analytical records, we confirm that the correct final analytical sample size is 1478, not 1024. The value of 1024 was an inadvertent carryover from a prior manuscript and was incorrectly retained in the present text. Table 1, which reports n = 1478, accurately reflects the analytical sample used in all statistical analyses. Accordingly, we have revised all in-text references to the sample size in the relevant sections to consistently report n = 1478. We believe this correction resolves the discrepancy and improves the overall clarity and internal consistency of the manuscript.

**Comment 6:** Furthermore, the path coefficient from EI to Relational SDL is reported as β = 1.093, which is unusually high for a standardized coefficient. This should be verified and reported correctly.

**Response to Comment 6:** We sincerely thank the reviewer for identifying this issue. The reviewer's concern is entirely well-founded: a standardized path coefficient of β = 1.093 would be statistically impossible, as standardized coefficients are bounded between −1 and +1 in standard regression frameworks. Upon auditing the full Amos output for both models, we identified that all path coefficients reported in Sections 3.4.1 and 3.4.2 — and displayed in Figures 3 and 4 — had been inadvertently drawn from the unstandardized regression weights matrix rather than the standardized regression weights matrix, a consistent error affecting both mediation models.

We have corrected Sections 3.4.1 and 3.4.2 accordingly, replacing all reported values with their corresponding standardized estimates confirmed from the Standardized Regression Weights output and cross-verified against the User-Defined Estimands table. In Section 3.4.1, the path coefficient from emotional intelligence to cognitive self-directed learning is now reported as β = 0.643 (p < 0.01), the direct path from emotional intelligence to clinical decision-making confidence as β = 0.133 (p < 0.01), and the path from cognitive self-directed learning to clinical decision-making confidence as β = 0.527 (p < 0.01). In Section 3.4.2, the path from emotional intelligence to relational self-directed learning is now reported as β = 0.601 (p < 0.01), the direct path from emotional intelligence to clinical decision-making confidence as β = 0.233 (p < 0.01), and the path from relational self-directed learning to clinical decision-making confidence as β = 0.401 (p < 0.01). Figures 3 and 4 have been updated in parallel, with all path coefficients — including the indicator loadings within each latent variable — replaced by their standardized values to ensure complete consistency throughout the diagrams. These corrections ensure that all reported path coefficients are statistically coherent and conform to established SEM reporting conventions.

**Fig 3 Structural equation model of cognitive self-directed learning in emotional intelligence and clinical decision-making confidence in nurses (n=1478)**

**Fig 4 Structural equation model of relational self-directed learning in emotional intelligence and clinical decision-making confidence in nurses (n=1478)**

**Comment 7:** The finding of a complete absence of family/carer involvement in the reviewed studies is a striking indicator of a gap in person-centered care within prison health and deserves to be a central point of the discussion. The institutional and security-related barriers that likely cause this omission should be discussed, emphasizing why this represents a critical failure of person-centered care in prisons.

**Comment 8:** The most striking finding, the complete absence of measures related to family and carer involvement, deserves to be elevated to a central point of the discussion, perhaps in its own paragraph. The institutional and security-related barriers that likely cause this gap should be discussed, emphasizing why its absence represents a critical failure of person-centred care in prisons.

**Comment 9:** The discussion on power imbalances and lack of autonomy is excellent. To strengthen the theoretical framing, these issues should be more explicitly linked to established concepts like "structural stigma" or "coercive environments" that are intrinsic to the carceral setting and fundamentally shape patient-provider interactions.

**Comment 10:** Please confirm and standardize the count of survey-based studies throughout the manuscript. The abstract states 21 studies (52%) utilized surveys, while the body mentions 24 studies. The correct number must be uniformly applied.

**Comment 11:** The call for new tools is clear and would be strengthened by explicitly recommending their co-design with people who have lived experience of incarceration. This aligns with participatory research paradigms and is crucial for ensuring the resulting tools are contextually valid and meaningful.

**Comment 12:** While limitations are acknowledged, the discussion could more directly address how the focus on explicit patient experience measures in titles/abstracts may have omitted relevant data embedded in broader studies of health in prisons.

**Comment 13:** The conclusion succinctly summarizes the review's contributions. The final sentence could be more powerful by directly echoing the primary recommendation, for example: "...to address health inequities faced by people experiencing incarceration through the co-design, validation, and implementation of context-specific patient experience measures."

**Response to Comment 7-13:** We sincerely appreciate the reviewer's time and scholarly investment in providing this series of comments. Upon reviewing Comments 7 through 13, it appears that the issues raised—such as family involvement in prison health, carceral contexts, and systematic review methodologies—do not pertain to the present study.Our manuscript is a primary, cross-sectional investigation focused on emotional intelligence, self-directed learning, and clinical decision-making among nurses in hospital settings in China. It does not involve incarcerated populations or prison health systems.We suspect that these comments may have been intended for a different manuscript under review. We have brought this to the editor's attention for clarification. We remain grateful for the reviewer's engagement and expertise.

**Comment 14:** The statement on page 35 that self-directed learning has a "58% total mediation rate" (33.9% + 24.1%) is a statistical misinterpretation. The two mediators are correlated components of the same broader construct (SDL), and their effects are not additive. The discussion should focus on comparing the two pathways rather than summing their effects.

**Response to Comment 14:** We are grateful to the reviewer for identifying this statistical misinterpretation. We wish to note that a substantively identical concern was raised independently by Reviewer 5 (Comment 8) and Reviewer 6 (Comment 7), and the present revision addresses all three sets of feedback through a unified and comprehensive correction.

The previously reported aggregate figure of 58% — calculated by summing the two separately estimated mediation proportions of 33.9% and 24.1% — has been fully removed from the manuscript. As the reviewer correctly notes, the two mediating pathways were estimated within structurally independent models (Figures 3 and 4 respectively), and because the two proportions derive from separate total-effect denominators, their simple addition does not yield a meaningful combined estimate. Accordingly, in Sections 3.5 and 4.2, the mediation results are now reported exclusively in comparative terms, with the proportion attributed to cognitive self-directed learning stated as 71.8% of its model's total standardized effect and that attributed to relational self-directed learning stated as 50.8% of its model's total standardized effect — figures that themselves reflect a separate correction to the Total row in Table 3, where unstandardized values had been inadvertently mixed with standardized path coefficients (detailed in our responses to Reviewer 5 Comment 8 and Reviewer 6 Comment 7). In Section 4.3, the discussion likewise presents the two proportions separately and frames them in explicitly comparative language, foregrounding the differential mediating strength of the two pathways rather than any aggregated sum. We also revised the Abstract and Conclusion to ensure that neither the erroneous 58% figure nor any analogous additive formulation appears elsewhere in the manuscript. These corrections substantially improve the statistical accuracy and interpretive coherence of the mediation findings.

**Comment 15:** The cross-sectional design is noted as a limitation, but the language in the results and discussion often implies causality (e.g., "emotional intelligence positively influenced..."). The manuscript must consistently use correlational language (e.g., "was associated with," "predicted") to avoid causal claims.

**Response to Comment 15:** We sincerely thank the reviewer for this careful observation. We fully agree that causal language is inappropriate given the cross-sectional design of this study, and we have conducted a thorough review of the manuscript to address this concern systematically. We note that a closely related concern was raised by Reviewer 6 (Comment 2), and both sets of feedback have been addressed through a coordinated set of revisions applied consistently across the manuscript.Expressions such as "emotional intelligence directly strengthens clinical decision-making confidence," "serves as a fundamental catalyst," and "creates optimal conditions...which subsequently builds" were replaced with correlational constructions including "is positively associated with," "may serve as a significant predictor of," and "suggests a potential pathway." Throughout the Discussion, language that attributed active causal mechanisms to emotional intelligence — such as assertions that it "transforms emotional awareness into clinical competence" or "demonstrates how emotional intelligence satisfies psychological needs" — was revised to employ modal constructions ("may suggest," "may facilitate," "could support," "are consistent with") that appropriately convey the interpretive and exploratory nature of the proposed mechanisms. These revisions have been applied consistently across the Results and Discussion sections to ensure that all inferential claims remain aligned with what a cross-sectional design can responsibly support, avoiding any overstatement of directionality or causal strength in the observed associations.

**Comment 16:** There is a significant chronological inconsistency: the manuscript states data was collected "between June and August 2025," which is in the future. Furthermore, several references are from 2024 and 2025. This must be corrected to reflect the actual data collection timeline, or the status of the 2025 references must be clarified (e.g., "Advance online publication").

A number of recent, contextually relevant studies support the core themes of this manuscript and can be used to strengthen the scholarly grounding.

-In the Introduction and Discussion, the work of Aboalrob et al. (2025), "Understanding the influence of self-concept on clinical decision-making among nurses" (PLOS ONE), provides direct, independent evidence for studying psychological predictors of CDMC, helping to position your focus on EI within a broader nomological network.

-The study by Ayed et al. (2025), "Insights into the relationship between emotional intelligence and critical thinking among nursing students" (BMC Nursing), offers robust external validation for your Hypothesis 2 (EI -> SDL), as critical thinking is a core component of cognitive self-directed learning. This should be cited when developing your rationale or interpreting this finding.

-The findings of Batran et al. (2025), "The relationship between professional quality of life and work environment among nurses..." (PLOS ONE), help contextualize your "relational SDL" pathway by illustrating how the professional environment and interpersonal dynamics are critically linked to nurse outcomes. This supports the discussion of how relational factors impact confidence.

-The paper by Aqtam et al. (2025), "Work engagement and its association with emotional intelligence..." (PLOS ONE), reinforces the significance of EI as a valuable construct in the nursing profession, linking it to positive work-related states and supporting the overall focus of your study.

These references provide clear and strong support for the theoretical framing, methodological considerations, and practical implications of the study and should be incorporated to enhance the manuscript's scholarly impact.

Suggested reference list

1.	Aboalrob, W., Ayed, A., Malak, M. Z., & Aqtam, I. (2025). Understanding the influence of self-concept on clinical decision-making among nurses: A cross-sectional study. PLOS ONE, 20(8), e0330905. https://doi.org/10.1371/journal.pone.0330905

2.	Ayed, A., Aqtam, I., Malak, M. Z., et al. (2025). Insights into the relationship between emotional intelligence and critical thinking among nursing students. BMC Nursing, 24, 1107. https://doi.org/10.1186/s12912-025-03782-7

3.	Batran, A., Aqtam, I., Ayed, A., & Ejheisheh, M. A. (2025). The relationship between professional quality of life and work environment among nurses in neonate care units. PLOS ONE, 20(4), e0322023. https://doi.org/10.1371/journal.pone.0322023

4.	Aqtam I, Ayed A, Batran A, Ejheisheh MA, Melhem RH, Shouli M (2025) Work engagement and its association with emotional intelligence and demographic characteristics among nurses in Palestinian neonatal intensive care units. PLoS One 20(9): e0332908. <https://doi.org/10.1371/journal.pone.0332908>

**Response to Comment 16:** We are grateful to the reviewer for raising these concerns and for the thoughtful suggestion of additional references. We address each issue in turn.

Regarding the reported chronological inconsistency, we wish to clarify that the data collection period stated in the manuscript accurately reflects the actual timeline of the study. All four references from 2025 recommended by the reviewer — Aboalrob et al., Ayed et al., Batran et al., and Aqtam et al. — were formally published in peer-reviewed journals prior to the submission of this manuscript, and their inclusion therefore does not constitute a chronological inconsistency. We have confirmed the publication status of each reference against the official journal records and are confident that all citations are appropriately dated.

Regarding the suggested references, we thank the reviewer for these highly relevant recommendations, all four of which have been incorporated into the manuscript. In the Introduction, Aboalrob et al. (2025) is cited in the conceptual framing of clinical decision-making confidence to position nursing self-concept as a core psychological predictor, situating the present study's focus on emotional intelligence within a broader nomological network of professional psychological constructs. Ayed et al. (2025) is cited in the development of Hypothesis 2 to provide external validation for the proposed association between emotional intelligence and cognitive self-directed learning, given that critical thinking represents a constitutive component of that learning dimension. In the Discussion, Section 4.1.1 incorporates Aboalrob et al. (2025) to deepen the interpretation of learning awareness as a mechanism of professional identity construction rather than mere knowledge accumulation, strengthening the argument that the psychological dimension of self-directed learning may be a more robust predictor of decision-making confidence than informational acquisition alone. Section 4.1.2 draws on Aqtam et al. (2025) to extend the interpretation of emotional utilization beyond situational awareness to encompass work engagement — characterized by vigor and dedication — as an affective foundation for clinical confidence.Section 4.2 integrates Ayed et al. (2025) within the cognitive mediation discussion to provide a mechanistic account of the cognitive pathway's predominance via emotional intelligence's association with reduced cognitive load, and incorporates Batran et al. (2025) within the relational mediation discussion to ground the interpersonal pathway in the established relationship between collegial collaboration, compassion satisfaction, and professional outcomes.

These additions collectively strengthen the theoretical grounding and empirical contextualization of both the Introduction and Discussion sections.

**Dear Reviewer #5:**

Thank you for your helpful suggestions on our manuscript. We have carefully considered all your comments and revised our manuscript accordingly. *Revised portion are marked in red in Revised Manuscript with Track Changes.* Below are our point-by-point responses to your comments:

**Comment 1:** Mean should be calculated by dividing the total score by the number of items. In this way, for example, when using a 5-point Likert scale, the mean score will range from 1 to 5, which allows for an intuitive interpretation of the magnitude of the mean. Accordingly, all related values and descriptions should be revised to reflect this calculation method.

**Comment 7:** The mean score should be calculated by dividing the total score by the number of items. The analyses should be rerun accordingly, as the values may differ slightly, and the updated values should be reported.

**Response to Comment 1 ＆ 7:** We thank the reviewer for this methodologically important observation regarding score calculation. We fully agree that item-average scores, derived by dividing total scale scores by the number of items, afford more intuitive interpretation by anchoring values within the original Likert scale range. In response to both comments, we revised section 3.2 of the Results to report item-average subscale scores in place of total scores for all three instruments. Specifically, subscale item averages on the Wong and Law Emotional Intelligence Scale (7-point scale), the Clinical Decision-Making Self-Confidence Scale (5-point scale), and the Self-Rating Scale of Self-Directed Learning (5-point scale) are now reported with their respective standard deviations, and the descriptive characterizations of performance levels have been updated accordingly. All reported values reflect the revised calculation method, and S4 Table has been comprehensively updated to incorporate item-average scores with the potential score range for each instrument explicitly noted. All relevant descriptions throughout the manuscript have likewise been updated to reflect this approach consistently.

**Descriptive analysis (n = 1478)**

| **Item** | **Total score ranges** | **Total scores (M ± SD)** | **Average scores (M ± SD)** |
| --- | --- | --- | --- |
| **Wong and Law Emotional Intelligence Scale** |  |  |  |
| Self-Emotion Appraisal | 10-28 | 24.49±3.49 | 6.12±0.87 |
| Others-Emotion Appraisal | 8-28 | 21.89±3.70 | 5.47±0.93 |
| Use of Emotion | 4-28 | 21.92±3.92 | 5.48±0.98 |
| Regulation of Emotion | 7-28 | 20.63±4.49 | 5.16±1.12 |
| **Clinical Decision-Making Self-Confidence Scale** |  |  |  |
| Identification | 6-15 | 12.23±1.71 | 4.08±0.57 |
| Assessment | 6-15 | 11.77±1.58 | 3.92±0.52 |
| Intervention | 6-15 | 11.91±1.59 | 3.97±0.53 |
| Evaluation | 7-15 | 11.69±1.60 | 3.90±0.53 |
| **Self-Rating Scale of Self-Directed Learning** |  |  |  |
| Learning Awareness | 34-60 | 48.14±4.96 | 4.01±0.41 |
| Learning Strategies | 30-60 | 47.59±5.63 | 3.97±0.47 |
| Learning Behavior | 28-60 | 45.72±6.40 | 3.81±0.53 |
| Learning Evaluation | 23-60 | 47.72±6.16 | 3.98±0.51 |
| Interpersonal Relationships | 33-60 | 48.04±6.00 | 4.00±0.50 |
| **Note:** The Wong and Law Emotional Intelligence Scale uses a 7-point Likert scale, whereas the Clinical Decision-Making Self-Confidence Scale and the Self-Rating Scale of Self-Directed Learning use 5-point Likert scales. Total score ranges reflect the observed minimum and maximum in this sample. M = mean; SD = standard deviation. Higher scores indicate higher levels of the corresponding construct. | | | |

**Comment 2:** In the Abstract, the phrase “with cognitive pathways demonstrating stronger mediation (33.9%) than relational pathways (24.1%)” is inappropriate, as these pathways were not compared within the same model.

**Response to Comment 2:** We appreciate the reviewer's identification of this concern, which bears directly on the inferential accuracy of the manuscript. The original phrase implied a direct cross-model comparison between two mediation pathways that were tested in separate structural equation models rather than estimated simultaneously within a single framework, which overstated the analytic basis for ranking the two pathways.

In response, we have revised the Abstract and Conclusion sections. In the revised Abstract, the Results subsection presents each mediation pathway independently, attributing the indirect effect and its proportion of the total standardized effect to its respective model without drawing comparative inferences across models. The revised Conclusion similarly characterizes the two pathways as findings from parallel models, describing their contributions in model-specific terms. These revisions more accurately represent the study's analytic structure and ensure that interpretive claims are bounded by the scope of the models from which they are derived.

**Comment 3:** The name of the mediating variable is inconsistent with the subsequent description. In Figure 4, it is labeled as learning evaluation, but it appears as relational self-directed learning in the Abstract. This inconsistency should be clarified and corrected.

**Response to Comment 3:** We thank the reviewer for identifying this inconsistency. The mislabeling in Figure 4, where the mediating variable was incorrectly displayed as "learning evaluation" rather than "Relational Self-Directed Learning," has been corrected in the revised manuscript. We also conducted a thorough review of all relevant sections to ensure that the terminology is used consistently throughout, including the Abstract, Section 3.4.2, and all associated figure captions. We appreciate the reviewer's careful attention, as this correction improves the clarity and terminological coherence of the manuscript.

**Fig 4 Structural equation model of relational self-directed learning in emotional intelligence and clinical decision-making confidence in nurses (n=1478)**

**Comment 4:** In the Introduction, the research objectives should be presented before the hypotheses.

**Response to Comment 4:** We appreciate the reviewer's suggestion regarding the logical sequencing of the Introduction and agree that presenting the research objectives prior to the hypotheses better aligns with standard deductive structure, in which aims are established before theoretical predictions are stated. In response, we have restructured Section 1 accordingly. The three research objectives have been relocated to immediately precede the hypothesis development section, ensuring that the study's aims are clearly established before the specific directional hypotheses are introduced. The final sentence concerning practical implications remains at the close of the Introduction. We believe this restructuring enhances the readability and logical flow of the Introduction without altering any substantive content.

**Comment 5:** The hypotheses are also problematic. Only one mediation hypothesis is stated in the manuscript; however, two models are tested in the subsequent analyses, each examining a separate mediation effect. If two mediating variables (i.e., treating the two dimensions as separate variables) are assumed, it would be more appropriate to explicitly state two hypotheses, each proposing the existence of a mediation effect. If there are no prior empirical studies examining some of the direct pathways among the variables, this should be explicitly acknowledged and stated in the manuscript.

**Response to Comment 5:** We thank the reviewer for this important methodological observation. The original manuscript presented a single mediation hypothesis despite the analyses examining cognitive and relational self-directed learning as two separate mediating pathways, and we agree that this inconsistency needed to be addressed. In response, we have revised the Introduction to replace the original Hypothesis 4 with two explicit sub-hypotheses: Hypothesis 4a, proposing that cognitive self-directed learning mediates the relationship between emotional intelligence and clinical decision-making confidence, and Hypothesis 4b, proposing the same for relational self-directed learning (Figure 1). To address the reviewer's additional concern regarding the empirical basis for certain direct pathways, we have added a statement in the theoretical discussion explicitly acknowledging that systematic empirical investigation of cognitive and relational self-directed learning as independent mediating pathways remains limited, and that Hypotheses 4a and 4b therefore represent an exploratory extension of existing frameworks.

We additionally note that the empirical grounding of Hypotheses 4a and 4b is strengthened by the most recent available evidence in the field. In response to a related concern raised separately during the revision process, we have incorporated Aboalrob et al. (2025) and Ayed et al. (2025) to reinforce the evidentiary basis of the proposed individual mediating pathways. We further note that Reviewer 5, Comment 5 and Reviewer 6, Comment 1 independently raised overlapping concerns regarding the theoretical grounding of the relational mediation pathway; the revisions developed in response to these respective comments have accordingly been designed in an integrated manner to ensure conceptual coherence across the manuscript. The theoretical elaboration provided in our response to Reviewer 6, Comment 1 — which articulates the mechanism linking emotional intelligence to relational self-directed learning through Self-Determination Theory and Experiential Learning Theory — provides further conceptual grounding for Hypothesis 4b and is therefore complementary to the structural revisions described here.

**Figure 1 Model Hypothesis Diagram**

**Comment 6:** The description of the Self-Rating Scale of Self-Directed Learning (SRSSDL) should be more concise and clearly structured. As currently presented, it is unclear how many dimensions the scale includes, whether it consists of multiple subscales, or how many dimensions are contained within each subscale. This needs to be clarified to avoid confusion.

**Response to Comment 6:** We thank the reviewer for this constructive observation, which has prompted a meaningful improvement in the clarity and structural precision of the manuscript.

In response to this comment, we have substantially revised Section 2.4.3. The original description did not explicitly name the five dimensions of the SRSSDL, and the justification for regrouping them into two subscales relied on Garrison's theoretical framework, which—as we recognized upon reflection—was not referenced elsewhere in the manuscript and therefore introduced an unnecessary conceptual discontinuity. In the revised version, we first enumerate all five original dimensions of the SRSSDL to establish a transparent baseline. We then ground the rationale for the two-subscale structure in the dimensional distinctions proposed by Yue et al., a reference already integrated into the theoretical framework of the introduction, thereby ensuring logical consistency across the manuscript. The revised text explicitly identifies the Cognitive Self-Directed Learning subscale (C-SDL) as comprising 48 items across four dimensions and the Social and Relational Self-Directed Learning subscale (SR-SDL) as comprising 12 items from the Interpersonal Relationships dimension. We believe these revisions directly address the reviewer's concern by making the scale structure, subscale composition, and item distribution unambiguous to the reader.

**Comment 8:** The value reported as the total effect is confusing. As it appears to represent the sum of the direct and indirect effects, and all effects are reported as positive, the total effect should not be smaller than either component. The authors should clarify how the total effect was calculated and verify whether the reported values are correct.

**Response to Comment 8:** We sincerely thank the reviewer for identifying this inconsistency, which reflects a genuine reporting error that we have now fully corrected.

The original error arose from a mixing of standardized and unstandardized outputs from the Amos bootstrap analysis. Specifically, all individual path coefficients in Table 3 (stda, stdb, stdc, and stdIndA1) were drawn from Amos's standardized bootstrap output; however, the Total row was inadvertently populated with values from the unstandardized "Total Effects" matrix (Estimate = 0.203 for both models, SE = 0.018, 95% CI [0.168, 0.239]). Because unstandardized and standardized solutions operate on different scales, the Total entry appeared smaller than the indirect effect alone—precisely the anomaly the reviewer correctly flagged.

We have corrected Table 3 in Section 3.5 by replacing the Total row with values drawn from Amos's Standardized Total Effects bootstrap output (bias-corrected percentile method). The corrected standardized total effect of emotional intelligence on clinical decision-making confidence is 0.472 (SE = 0.034, 95% CI [0.403, 0.536], p < 0.01) for Model 1 and 0.474 (SE = 0.034, 95% CI [0.404, 0.537], p < 0.01) for Model 2, both of which are appropriately larger than their respective indirect effects (0.339 and 0.241). Consequently, the mediation proportions reported in Sections 3.5 and 4.2 have also been updated: the figure for cognitive self-directed learning has been corrected from 33.9% to 71.8% (= 0.339/0.472), and the figure for relational self-directed learning from 24.1% to 50.8% (= 0.241/0.474). We also note that the aggregate "58% total mediation rate" cited in Section 4.3—which was the sum of the two incorrectly computed proportions—has been removed, as summing mediation proportions across two structurally independent models does not yield a meaningful combined estimate; the two corrected proportions are now reported separately in that section. We believe these corrections substantially improve the accuracy and internal consistency of the mediation findings throughout the manuscript.

**Table 3 Bootstrap Test of mediation effect (n=1478)**

| **Model** | **Pathway** | **SE** | **Estimate** | **Lower** | **Upper** | ***p* Value** |
| --- | --- | --- | --- | --- | --- | --- |
| **Model 1** | Emotional Intelligence → Cognitive Self-Directed Learning | 0.028 | 0.643 | 0.585 | 0.698 | ＜0.01** |
|  | Cognitive Self-Directed Learning → Confidence In Clinical Decision-Making | 0.047 | 0.527 | 0.431 | 0.617 | ＜0.01** |
|  | Emotional Intelligence → Confidence In Clinical Decision-Making | 0.049 | 0.133 | 0.036 | 0.231 | 0.01* |
|  | Emotional Intelligence → Cognitive Self-Directed Learning → Confidence In Clinical Decision-Making | 0.034 | 0.339 | 0.273 | 0.409 | ＜0.01** |
|  | Total | 0.034 | 0.472 | 0.403 | 0.536 | ＜0.01** |
| **Model 2** | Emotional Intelligence → Relational Self-Directed Learning | 0.028 | 0.601 | 0.543 | 0.655 | ＜0.01** |
|  | Relational Self-Directed Learning → Confidence In Clinical Decision-Making | 0.045 | 0.401 | 0.311 | 0.49 | ＜0.01** |
|  | Emotional Intelligence → Confidence In Clinical Decision-Making | 0.049 | 0.233 | 0.133 | 0.326 | ＜0.01** |
|  | Emotional Intelligence → Relational Self-Directed Learning → Confidence In Clinical Decision-Making | 0.03 | 0.241 | 0.185 | 0.302 | ＜0.01** |
|  | Total | 0.034 | 0.474 | 0.404 | 0.537 | ＜0.01** |
| **p*＜0.05, ***p*＜0.01 | | | | | | |

**Dear Reviewer #6:**

Thank you for your helpful suggestions on our manuscript. We have carefully considered all your comments and revised our manuscript accordingly. *Revised portion are marked in red in Revised Manuscript with Track Changes.* Below are our point-by-point responses to your comments:

**Comment 1:** Theoretical framework and mechanism explanation

Problem: Although the research mentions self-determination theory and experiential learning theory, there is insufficient explanation on how they specifically support the path mechanism of "emotional intelligence → autonomous learning → decision-making confidence". The theoretical foundation of the "relationship path" in the model is weak (such as how interpersonal relationships can be transformed into learning motivation?).

**Response to Comment 1:** We are grateful to the reviewer for identifying this theoretical gap and agree that both the overarching path mechanism and the relational pathway in particular warranted more explicit theoretical development. We note that Reviewer 5, Comment 5 raised a closely related concern regarding the grounding of the mediation hypotheses, and the restructuring of the hypothesis section described in our response to that comment — including the addition of explicit sub-hypotheses and an acknowledgment of the exploratory nature of the relational pathway — complements the theoretical revisions detailed here (Figure 1).

In response to the present comment, we have substantially revised the theoretical framework section to address both dimensions of the reviewer's concern. Regarding the overarching pathway, the revised passage clarifies how Self-Determination Theory and Experiential Learning Theory jointly support the "emotional intelligence → autonomous learning → decision-making confidence" mechanism: high-EI practitioners are better positioned to internalize autonomous motivation for learning and to engage productively in the reflective observation cycles through which clinical knowledge is consolidated into actionable confidence. Regarding the relational pathway specifically, the revised passage clarifies, through the lens of Self-Determination Theory, that the interpersonal dimensions of emotional intelligence facilitate satisfaction of the relatedness need, which in turn strengthens autonomous motivation for collaborative and observationally based learning. We additionally draw on Aqtam et al. (2025), who demonstrate that emotionally intelligent nurses translate social competence and emotion utilization into heightened work engagement, providing an empirically grounded motivational account of how interpersonal engagement is transformed into learning behavior. The treatment of Experiential Learning Theory has been extended to specify that peer dialogue and interpersonal exchange constitute primary vehicles for reflective observation, offering a more complete and mechanistically explicit theoretical foundation for the relational pathway.

**Figure 1 Model Hypothesis Diagram**

**Comment 2:** Limitations of Causal Inference

Problem: The cross-sectional design cannot confirm causality, but the manuscript implies causality in multiple places (such as "emotional intelligence catalyzes learning behavior"). The full text should clearly emphasize the essential relevance of the research and avoid overinterpreting causality.

**Response to Comment 2:** We appreciate the reviewer for raising this important concern regarding the inferential boundaries of the cross-sectional design. We note that Reviewer 4 (Comment 15) identified the same issue, and the revisions described here reflect a coordinated response to both sets of feedback. A systematic review of the Introduction and Discussion sections was undertaken to identify and revise all instances in which the manuscript's language implied causal directionality beyond what the study design can support. Causal constructions — including phrases such as "emotional intelligence catalyzes learning behavior," "directly enhances clinical decision-making confidence," and "the superiority of this pathway reflects...mechanisms that convert emotional resources into decision certainty" — were replaced with language that accurately characterizes the associations observed. Revised expressions include "is positively associated with," "may reduce," "potentially associated with," "may be better positioned to," and "are consistent with the theoretical framework proposed through." Modal verbs have been introduced systematically throughout the mechanistic discussion to convey that the proposed cognitive and relational pathways represent theoretical interpretations consistent with the observed associations, rather than empirically confirmed causal sequences. We believe these revisions strengthen the scholarly integrity of the manuscript by ensuring that the framing of all findings reflects appropriate epistemic caution, preserving the substantive contribution of the study while avoiding any overinterpretation of its findings.

**Comment 3:** Insufficient theoretical basis for the restructuring of scale dimensions

The author reorganized the original five factor structure of SRSSDL into two dimensions: cognitive autonomous learning (C-SDL) and social relationship autonomous learning (SR-SDL). Although the Garrison model is cited as theoretical support, has this recombination been tested by confirmatory factor analysis (CFA)? Is the CFA result reported in the article (lines 293-295) for the recombined two factor model or the original five factor model? Suggest the author to provide the comparison results between the two factor model and the original five factor model (such as chi square difference test, AIC/BIC comparison) to demonstrate the rationality of the recombination.

**Response to Comment 3:** We are grateful for the reviewer's observation and fully agree that the structural validity of the proposed dimensional reorganization requires more explicit justification. This point is well taken, and we have revised Section 2.4.3 accordingly.

We would like to first clarify a potential source of ambiguity in the original manuscript: the CFA indices reported in lines 293–295 (χ²/df = 2.255, CFI = 0.856, TLI = 0.850, RMSEA = 0.035, SRMR = 0.039) reflected the five-factor structure of the SRSSDL rather than the proposed two-factor reorganization. We recognize that this was not made sufficiently clear and apologize for any confusion this may have caused.

In response to the reviewer's suggestion, we conducted a comparative CFA contrasting the original five-factor solution with the proposed two-factor structure, and the CFA indices for the two-factor model are now reported in the revised Section 2.4.3. For reference, the full comparison is as follows: the five-factor model yielded CFI = 0.856, TLI = 0.850, RMSEA = 0.035, SRMR = 0.039, AIC = 113917.24, and BIC = 114854.22, while the two-factor model produced CFI = 0.803, TLI = 0.796, RMSEA = 0.041, SRMR = 0.043, AIC = 114695.33, and BIC = 115587.93. A chi-square difference test indicated a statistically significant improvement in fit for the five-factor solution (Δχ² = 796.09, Δdf = 9, p < .001). We acknowledge that the two-factor model does not outperform the original five-factor structure on these indices. However, we would respectfully note that the absolute fit indices of the two-factor model — RMSEA = 0.041 and SRMR = 0.043 — remain within generally accepted thresholds, and that neither model reaches the conventional CFI benchmark of 0.90, a pattern that may reflect the inherent complexity of the 60-item scale rather than a fundamental misspecification.

We would also like to humbly emphasize that the two-factor reorganization was motivated primarily by the theoretical requirements of the research design rather than by a pursuit of improved statistical fit. The core hypotheses of the present study concern the differential mediating roles of cognitive and social-relational self-directed learning pathways, a distinction that necessitates the operationalization of these mechanisms as separable constructs. In this sense, the two-factor structure is a theoretically driven operationalization that is grounded in the framework proposed by Yue et al. (2025), and its acceptable absolute fit is considered sufficient to support the proposed mediation analyses. We hope that these clarifications and the added comparative evidence address the reviewer's concern, and we remain open to further discussion should any ambiguity persist.

**Comment 4:** Sample representativeness and universality

Question: Women account for 97.56% of the sample, mainly from tertiary hospitals, which limits the generalizability of the conclusion to male nurses and primary healthcare institutions. In addition, women accounted for 97.56% of the sample, while tertiary hospitals accounted for 97.70%. This highly homogeneous sample may limit the extrapolation of research conclusions. The author has mentioned this issue in the Limitations section, but suggests discussing in more detail how these features may affect research findings in the Discussion.

**Response to Comment 4:** We appreciate the reviewer's recommendation that the implications of sample homogeneity be discussed in greater detail with respect to how these characteristics may specifically affect the research findings. As noted in the response to Reviewer 4 Comment 3 above, we have added Section 4.1.4 (Impact of Sample Homogeneity on Confidence Patterns) to address this concern directly. The new section explicitly discusses how the concentration of tertiary hospital participants may have shaped the observed confidence levels and departmental variation, and how the near-exclusive representation of female nurses may have influenced the strength of the emotional utilization predictor in the regression model, thereby providing the more substantive interpretive engagement that the reviewer recommended.

**Comment 5:** Data sources and measurement deviations

Problem: Relying on self-reported data may lead to social expectation bias. Clinical decision-making confidence is a subjective evaluation and is not associated with objective clinical manifestations.

**Response to Comment 5:** Thank you for this valuable observation. We fully agree that reliance on self-reported data constitutes a meaningful methodological limitation, and we appreciate the reviewer's specific point that clinical decision-making confidence, as a subjective construct, was not corroborated by objective clinical performance indicators. In response, we have revised Section 5 (Limitations and prospects) to address these concerns more explicitly. Limitation (3) has been expanded to acknowledge the risk of social desirability bias inherent in self-report instruments and to note that the subjective nature of clinical decision-making confidence, evaluated without reference to objective clinical performance, may constrain the criterion validity of this measure. The corresponding future research directions have been updated accordingly to underscore the value of incorporating objective performance-based assessments in mitigating these biases. We believe these revisions more transparently communicate the methodological constraints of the study and contribute to a more rigorous and balanced presentation of the findings.

**Comment 6:** The description of the sampling method does not match the actual operation

The author claims to use 'simple random sampling', but the actual process described in the methods section (distributed by nursing department coordinators, recruited during shift changes) is more in line with the characteristics of convenience sampling or cluster sampling. Simple random sampling requires randomly selecting each individual from a complete sampling box. Please clarify the actual sampling process or modify the description of the sampling method.

**Response to Comment 6:** We appreciate the reviewer's careful attention to methodological accuracy and acknowledge that this observation identifies a meaningful inconsistency in the original manuscript. The reviewer is correct that the recruitment procedures described—distribution through nursing department coordinators and participant contact during shift changes—are characteristic of convenience sampling rather than simple random sampling, which requires random selection from a complete sampling frame. We have revised Section 2.2 accordingly. The opening of the first paragraph now correctly identifies convenience sampling as the method employed and includes a concise statement acknowledging both the practical advantages of this approach and its inherent limitation with respect to potential selection bias. All prior references to simple random sampling and its associated methodological rationale have been removed. This correction more accurately represents the study's design and improves the transparency of the methods reporting.

**Comment 7:** The calculation of the percentage of mediation effect needs to be explained

The author reported that cognitive pathways mediated 33.9% and relational pathways mediated 24.1%, totaling 58% of the mediating effect. However, the direct effects in the Bootstrap analysis table (Table 3) were numerically different between the two models (Model 1: 0.133 vs Model 2: 0.233), suggesting that the two paths may have been estimated separately in different models. Can the author explain whether a complete model has been established that includes both mediation paths simultaneously? If two paths are estimated separately in different models, the simple addition of the two mediation effect percentages may be problematic.

**Response to Comment 7:** We sincerely thank the reviewer for this careful and incisive observation regarding the mediation proportion calculations. This comment, considered alongside a parallel concern raised by Reviewer 5 (Comment 8), prompted a thorough audit of the bootstrap outputs in Table 3, during which a substantive reporting error was identified and fully corrected.

The error originated from an inadvertent mixing of standardized and unstandardized outputs from the Amos bootstrap analysis. The Total row in Table 3 had been populated with unstandardized total effect values, while all individual path coefficients were drawn from the standardized solution — a discrepancy that accounts for the anomalous pattern the reviewer observed, wherein the total effect appeared numerically smaller than the indirect effect alone. We have corrected Table 3 in Section 3.5 by replacing the Total row with values derived from Amos's standardized total effects bootstrap output (bias-corrected percentile method). Consequently, the mediation proportions reported in Sections 3.5 and 4.2 have been updated: the proportion attributed to cognitive self-directed learning has been revised from 33.9% to 71.8% (= 0.339/0.472), and that attributed to relational self-directed learning from 24.1% to 50.8% (= 0.241/0.474).

With respect to the reviewer's question about model structure: the two mediation paths were estimated in structurally independent models rather than a single parallel model, as depicted in Figures 3 and 4, respectively. Given this design, the previously reported aggregate figure of 58% — obtained by summing proportions across two independent models — does not constitute a meaningful combined estimate, and we acknowledge this as an error in reporting. This figure has been removed from Section 4.3, and the two proportions are now reported separately. These corrections improve the accuracy and internal consistency of the mediation findings throughout the manuscript.

**Table 3 Bootstrap Test of mediation effect (n=1478)**

| **Model** | **Pathway** | **SE** | **Estimate** | **Lower** | **Upper** | ***p* Value** |
| --- | --- | --- | --- | --- | --- | --- |
| **Model 1** | Emotional Intelligence → Cognitive Self-Directed Learning | 0.028 | 0.643 | 0.585 | 0.698 | ＜0.01** |
|  | Cognitive Self-Directed Learning → Confidence In Clinical Decision-Making | 0.047 | 0.527 | 0.431 | 0.617 | ＜0.01** |
|  | Emotional Intelligence → Confidence In Clinical Decision-Making | 0.049 | 0.133 | 0.036 | 0.231 | 0.01* |
|  | Emotional Intelligence → Cognitive Self-Directed Learning → Confidence In Clinical Decision-Making | 0.034 | 0.339 | 0.273 | 0.409 | ＜0.01** |
|  | Total | 0.034 | 0.472 | 0.403 | 0.536 | ＜0.01** |
| **Model 2** | Emotional Intelligence → Relational Self-Directed Learning | 0.028 | 0.601 | 0.543 | 0.655 | ＜0.01** |
|  | Relational Self-Directed Learning → Confidence In Clinical Decision-Making | 0.045 | 0.401 | 0.311 | 0.49 | ＜0.01** |
|  | Emotional Intelligence → Confidence In Clinical Decision-Making | 0.049 | 0.233 | 0.133 | 0.326 | ＜0.01** |
|  | Emotional Intelligence → Relational Self-Directed Learning → Confidence In Clinical Decision-Making | 0.03 | 0.241 | 0.185 | 0.302 | ＜0.01** |
|  | Total | 0.034 | 0.474 | 0.404 | 0.537 | ＜0.01** |
| **p*＜0.05, ***p*＜0.01 | | | | | | |

**Comment 8:** Testing for common method bias

There is a risk of common method bias when collecting data using three self-assessment scales at the same time point in the study. Suggest the author report the results of Harman single factor test or other common method bias tests.

**Response to Comment 8:** We sincerely appreciate the reviewer's constructive attention to this methodological concern. The risk of common method bias in single time-point, self-report study designs is a legitimate and important consideration, and we agree that it warrants explicit reporting.

In response, we have revised Section 2.6 to clarify that R was utilized for reliability and validity assessments, and to describe the common method factor (CMF) analysis procedure adopted to evaluate potential common method bias. A concise results statement has been inserted between Section 3 (Results) and Section 3.1 (Participants' Sociodemographic Characteristics), reporting that the average method factor explained 15.03% of the total variance, which is well below the recommended 25% threshold, indicating that common method bias does not pose a meaningful threat to the validity of the study's findings. In addition, we have added clarification in Section 2.6 that, given the known sensitivity of the χ2/df statistic in large samples, a relaxed threshold of less than 5.0 was adopted in evaluating model fit, consistent with established methodological guidelines. We trust that these revisions adequately address the reviewer's concern and strengthen the overall methodological transparency of the manuscript.

**Comment 9:** Complete report of confirmatory factor analysis

The CFA results for three scales were reported in the article, but specific values for factor loadings were missing. Suggest providing complete CFA results in the supplementary materials, including standardized factor loadings and AVE/CR values.

**Response to Comment 9:** We sincerely appreciate the reviewer's professional insight regarding the reporting of confirmatory factor analysis (CFA) results. We agree that providing detailed item-level parameters is essential for demonstrating the construct validity of our instruments. In response, we have updated Sections 2.4.2, 2.4.3, and 2.4.4 to include the ranges for standardized factor loadings, composite reliability (CR), and average variance extracted (AVE) based on the full study sample. Furthermore, we have added S2 Table to the Supporting Information, which provides a comprehensive item-by-item breakdown of these psychometric parameters for the WLEIS, SRSSDL, and CDMSCS. Regarding the AVE values, we have included an explanatory note in S2 Table and addressed this in the manuscript text. While several dimensions (particularly within the 60-item SRSSDL) showed AVE values below the conventional 0.50 threshold, indicating modest convergent validity, the corresponding CR values remained at acceptable levels. In nursing education research, it is recognized that scales with a high number of items per dimension can exhibit lower AVE values while maintaining strong internal consistency. Given the adequate CR levels and the necessity of maintaining the structural integrity of these established instruments, the constructs were retained to preserve theoretical completeness. These revisions provide the transparency requested by the reviewer and strengthen the statistical foundation of our findings.

**S2 Table Standardized Factor Loadings, Composite Reliability (CR), and Average Variance Extracted (AVE) of All Measurement Instruments**

| **Scale** | **Dimension** | **Item** | **Standardized Loading** | **CR** | **AVE** |
| --- | --- | --- | --- | --- | --- |
| Wong and Law Emotional Intelligence Scale | Self-Emotion Appraisal | EI1 | 0.577 | 0.761 | 0.443 |
|  |  | EI2 | 0.720 |  |  |
|  |  | EI3 | 0.722 |  |  |
|  |  | EI4 | 0.657 |  |  |
|  | Others-Emotion Appraisal | EI5 | 0.571 | 0.689 | 0.357 |
|  |  | EI6 | 0.632 |  |  |
|  |  | EI7 | 0.566 |  |  |
|  |  | EI8 | 0.622 |  |  |
|  | Use of Emotion | EI9 | 0.613 | 0.720 | 0.391 |
|  |  | EI10 | 0.648 |  |  |
|  |  | EI11 | 0.644 |  |  |
|  |  | EI12 | 0.602 |  |  |
|  | Regulation of Emotion | EI13 | 0.680 | 0.795 | 0.492 |
|  |  | EI14 | 0.787 |  |  |
|  |  | EI15 | 0.768 |  |  |
|  |  | EI16 | 0.594 |  |  |
| Clinical Decision-Making Self-Confidence Scale | Identification | DC1 | 0.684 | 0.658 | 0.395 |
|  |  | DC2 | 0.708 |  |  |
|  |  | DC3 | 0.489 |  |  |
|  | Assessment | DC4 | 0.649 | 0.595 | 0.331 |
|  |  | DC5 | 0.580 |  |  |
|  |  | DC6 | 0.491 |  |  |
|  | Intervention | DC7 | 0.566 | 0.565 | 0.304 |
|  |  | DC8 | 0.535 |  |  |
|  |  | DC9 | 0.548 |  |  |
|  | Evaluation | DC10 | 0.634 | 0.614 | 0.348 |
|  |  | DC11 | 0.562 |  |  |
|  |  | DC12 | 0.571 |  |  |
| Self-Rating Scale of Self-Directed Learning | Learning Awareness | SDL1 | 0.453 | 0.736 | 0.190 |
|  |  | SDL2 | 0.496 |  |  |
|  |  | SDL3 | 0.394 |  |  |
|  |  | SDL4 | 0.458 |  |  |
|  |  | SDL5 | 0.510 |  |  |
|  |  | SDL6 | 0.534 |  |  |
|  |  | SDL7 | 0.548 |  |  |
|  |  | SDL8 | 0.578 |  |  |
|  |  | SDL9 | 0.279 |  |  |
|  |  | SDL10 | 0.347 |  |  |
|  |  | SDL11 | 0.511 |  |  |
|  |  | SDL12 | 0.363 |  |  |
|  | Learning Strategies | SDL13 | 0.368 | 0.780 | 0.233 |
|  |  | SDL14 | 0.349 |  |  |
|  |  | SDL15 | 0.390 |  |  |
|  |  | SDL16 | 0.464 |  |  |
|  |  | SDL17 | 0.563 |  |  |
|  |  | SDL18 | 0.498 |  |  |
|  |  | SDL19 | 0.616 |  |  |
|  |  | SDL20 | 0.538 |  |  |
|  |  | SDL21 | 0.571 |  |  |
|  |  | SDL22 | 0.417 |  |  |
|  |  | SDL23 | 0.497 |  |  |
|  |  | SDL24 | 0.494 |  |  |
|  | Learning Behavior | SDL25 | 0.522 | 0.807 | 0.271 |
|  |  | SDL26 | 0.521 |  |  |
|  |  | SDL27 | 0.514 |  |  |
|  |  | SDL28 | 0.468 |  |  |
|  |  | SDL29 | 0.520 |  |  |
|  |  | SDL30 | 0.546 |  |  |
|  |  | SDL31 | 0.548 |  |  |
|  |  | SDL32 | 0.478 |  |  |
|  |  | SDL33 | 0.584 |  |  |
|  |  | SDL34 | 0.596 |  |  |
|  |  | SDL35 | 0.455 |  |  |
|  |  | SDL36 | 0.313 |  |  |
|  | Learning Evaluation | SDL37 | 0.512 | 0.838 | 0.306 |
|  |  | SDL38 | 0.554 |  |  |
|  |  | SDL39 | 0.575 |  |  |
|  |  | SDL40 | 0.488 |  |  |
|  |  | SDL41 | 0.483 |  |  |
|  |  | SDL42 | 0.542 |  |  |
|  |  | SDL43 | 0.572 |  |  |
|  |  | SDL44 | 0.607 |  |  |
|  |  | SDL45 | 0.592 |  |  |
|  |  | SDL46 | 0.579 |  |  |
|  |  | SDL47 | 0.538 |  |  |
|  |  | SDL48 | 0.545 |  |  |
|  | Interpersonal Relationships | SDL49 | 0.527 | 0.803 | 0.257 |
|  |  | SDL50 | 0.491 |  |  |
|  |  | SDL51 | 0.582 |  |  |
|  |  | SDL52 | 0.535 |  |  |
|  |  | SDL53 | 0.516 |  |  |
|  |  | SDL54 | 0.472 |  |  |
|  |  | SDL55 | 0.486 |  |  |
|  |  | SDL56 | 0.477 |  |  |
|  |  | SDL57 | 0.488 |  |  |
|  |  | SDL58 | 0.534 |  |  |
|  |  | SDL59 | 0.466 |  |  |
|  |  | SDL60 | 0.502 |  |  |
| **Note:** All factor loadings were statistically significant (*p* <0.01). While most CR values met acceptable thresholds, several dimensions (particularly within SRSSDL) showed AVE values below the conventional criterion of 0.50, indicating modest convergent validity. Given the large number of items per dimension and adequate CR levels, the constructs were retained to preserve theoretical completeness. | | | | | |

**Comment 10:** In the author contribution statement on line 722, the third author's name is written as "Huan Ye", which is inconsistent with "Juan Ye" in other places. Please verify and unify. The p-value format in some tables is inconsistent (some use "<0.01 * *", some use "<0.01 * *"), it is recommended to use the standard format uniformly.

**Response to Comment 10:** We sincerely appreciate the reviewer's meticulous attention to detail in identifying these formatting and typographical inconsistencies. In response, we have corrected the typographical error in the CRediT authors' contributions section, ensuring the third author's name is consistently spelled as "Juan Ye" throughout the manuscript. Furthermore, we have thoroughly audited and standardized the p-value formatting across all applicable tables (Table 1, Table 2, and Table 3) and figure captions. The unified format now strictly employs an italicized p, includes single spaces on both sides of the mathematical operator, retains the leading zero before the decimal point, and places significance asterisks immediately following the numerical value without intervening spaces (e.g., *p* < 0.05*, *p* < 0.01**). We believe these rigorous standardizations substantially enhance the professional presentation, clarity, and typographical consistency of the final manuscript.
