# Supplementary material for: Enhancing nurses’ clinical decision-making confidence through dual pathways of self-directed learning: A structural equation model
Source: PLoS One. 2026 Jun 11;21(6):e0351551. doi: 10.1371/journal.pone.0351551 (PMC13257989; doi:10.1371/journal.pone.0351551)
Supplement: Peer review history

# Peer review: Decision Letter 0

**DOI:** 10.1371/journal.pone.0351551.r001

**Authors:** Sergio Consoli

4 Feb 2026

PONE-D-25-50160

Enhancing Nurses’ Clinical Decision-Making Confidence Through Dual Pathways of Self-Directed Learning: A Structural Equation Model

PLOS One

Dear Dr. Ye,

Thank you for submitting your manuscript to PLOS ONE. After careful consideration, we feel that it has merit but does not fully meet PLOS ONE’s publication criteria as it currently stands. Therefore, we invite you to submit a revised version of the manuscript that addresses the points raised during the review process.

Please submit your revised manuscript by Mar 20 2026 11:59PM. If you will need more time than this to complete your revisions, please reply to this message or contact the journal office at plosone@plos.org. When you're ready to submit your revision, log on to https://www.editorialmanager.com/pone/ and select the 'Submissions Needing Revision' folder to locate your manuscript file.

Please include the following items when submitting your revised manuscript:

- A letter that responds to each point raised by the academic editor and reviewer(s). You should upload this letter as a separate file labeled 'Response to Reviewers'.
- A marked-up copy of your manuscript that highlights changes made to the original version. You should upload this as a separate file labeled 'Revised Manuscript with Track Changes'.
- An unmarked version of your revised paper without tracked changes. You should upload this as a separate file labeled 'Manuscript'.

If you would like to make changes to your financial disclosure, please include your updated statement in your cover letter. Guidelines for resubmitting your figure files are available below the reviewer comments at the end of this letter.

If applicable, we recommend that you deposit your laboratory protocols in protocols.io to enhance the reproducibility of your results. Protocols.io assigns your protocol its own identifier (DOI) so that it can be cited independently in the future. For instructions see: https://journals.plos.org/plosone/s/submission-guidelines#loc-laboratory-protocols. Additionally, PLOS ONE offers an option for publishing peer-reviewed Lab Protocol articles, which describe protocols hosted on protocols.io. Read more information on sharing protocols at https://plos.org/protocols?utm_medium=editorial-email&utm_source=authorletters&utm_campaign=protocols.

We look forward to receiving your revised manuscript.

Kind regards,

Sergio Consoli

Academic Editor

PLOS One

Journal Requirements:

When submitting your revision, we need you to address these additional requirements.

1.Please ensure that your manuscript meets PLOS ONE's style requirements, including those for file naming. The PLOS ONE style templates can be found at

https://journals.plos.org/plosone/s/file?id=wjVg/PLOSOne_formatting_sample_main_body.pdf and

https://journals.plos.org/plosone/s/file?id=ba62/PLOSOne_formatting_sample_title_authors_affiliations.pdf

2. Please note that PLOS One has specific guidelines on code sharing for submissions in which author-generated code underpins the findings in the manuscript. In these cases, we expect all author-generated code to be made available without restrictions upon publication of the work. Please review our guidelines at https://journals.plos.org/plosone/s/materials-and-software-sharing#loc-sharing-code and ensure that your code is shared in a way that follows best practice and facilitates reproducibility and reuse.

3. PLOS requires an ORCID iD for the corresponding author in Editorial Manager on papers submitted after December 6th, 2016. Please ensure that you have an ORCID iD and that it is validated in Editorial Manager. To do this, go to ‘Update my Information’ (in the upper left-hand corner of the main menu), and click on the Fetch/Validate link next to the ORCID field. This will take you to the ORCID site and allow you to create a new iD or authenticate a pre-existing iD in Editorial Manager.

4. Please ensure that you include a title page within your main document. You should list all authors and all affiliations as per our author instructions and clearly indicate the corresponding author.

5. Your ethics statement should only appear in the Methods section of your manuscript. If your ethics statement is written in any section besides the Methods, please delete it from any other section.

6. Please remove your figures from within your manuscript file, leaving only the individual TIFF/EPS image files, uploaded separately. These will be automatically included in the reviewers’ PDF.

7. If the reviewer comments include a recommendation to cite specific previously published works, please review and evaluate these publications to determine whether they are relevant and should be cited. There is no requirement to cite these works unless the editor has indicated otherwise.

Additional Editor Comments:

In particular, the reviewers recognize merit in your work but identify critical issues requiring revision before acceptance:

1. **Methodological corrections:** Accurately describe sampling method (convenience/cluster, not simple random); resolve Table 1 sample size discrepancy; report common method bias testing.
2. **Statistical revisions:** Recalculate means as score/items and re-run analyses; verify unusual path coefficient; clarify total effect calculations.
3. **Measurement validation:** Provide CFA comparison justifying 2-factor vs. original 5-factor SRSSDL structure; include complete CFA results (factor loadings, AVE/CR) in supplementary materials.
4. **Hypothesis and interpretation:** State two separate mediation hypotheses; clarify that mediation percentages from separate models (33.9%, 24.1%) cannot be directly compared or summed.
5. **Language consistency:** Replace causal language with correlational terms throughout; correct variable labeling inconsistencies, chronological errors, and author name discrepancies.

Please also integrate recent literature; strengthen theoretical explanations; expand discussion of sample homogeneity limitations.

IMPORTANT NOTE TO AUTHORS:

Comments from the editorial office: Upon internal evaluation of the reviews, we kindly request you to disregard the reviewer reports provided by Reviewer 1 and 2. No amendments are required in response to comments of Reviewer 1 and 2.

[Note: HTML markup is below. Please do not edit.]

Reviewers' comments:

Reviewer's Responses to Questions

**Comments to the Author**

1. Is the manuscript technically sound, and do the data support the conclusions?

The manuscript must describe a technically sound piece of scientific research with data that supports the conclusions. Experiments must have been conducted rigorously, with appropriate controls, replication, and sample sizes. The conclusions must be drawn appropriately based on the data presented.

Reviewer #1: Yes

Reviewer #2: Partly

Reviewer #3: Yes

Reviewer #4: Partly

Reviewer #5: Partly

Reviewer #6: Partly

**********

2. Has the statistical analysis been performed appropriately and rigorously?

Reviewer #1: Yes

Reviewer #2: Yes

Reviewer #3: Yes

Reviewer #4: No

Reviewer #5: Yes

Reviewer #6: Yes

**********

3. Have the authors made all data underlying the findings in their manuscript fully available?

The PLOS Data policy requires authors to make all data underlying the findings described in their manuscript fully available without restriction, with rare exception (please refer to the Data Availability Statement in the manuscript PDF file). The data should be provided as part of the manuscript or its supporting information, or deposited to a public repository. For example, in addition to summary statistics, the data points behind means, medians and variance measures should be available. If there are restrictions on publicly sharing data—e.g. participant privacy or use of data from a third party—those must be specified.

Reviewer #1: Yes

Reviewer #2: Yes

Reviewer #3: Yes

Reviewer #4: No

Reviewer #5: Yes

Reviewer #6: Yes

**********

4. Is the manuscript presented in an intelligible fashion and written in standard English?

PLOS ONE does not copyedit accepted manuscripts, so the language in submitted articles must be clear, correct, and unambiguous. Any typographical or grammatical errors should be corrected at revision, so please note any specific errors here.

Reviewer #1: Yes

Reviewer #2: Yes

Reviewer #3: Yes

Reviewer #4: Yes

Reviewer #5: Yes

Reviewer #6: No

**********

5. Review Comments to the Author

Please use the space provided to explain your answers to the questions above. You may also include additional comments for the author, including concerns about dual publication, research ethics, or publication ethics. (Please upload your review as an attachment if it exceeds 20,000 characters)

Reviewer #1: Introduction

The introduction provides a strong theoretical rationale but would benefit from integrating recent international studies (2023–2025) addressing emotional intelligence, learning autonomy, and decision-making in nursing practice to enhance global relevance. Consider referencing studies such as:

Ayed, A. (2025). The relationship between the emotional intelligence and clinical decision-making among nurses in neonatal intensive care units. SAGE Open Nursing, 11, 23779608251321352.

Abu Arra, A. Y., Ayed, A., Toqan, D., Albashtawy, M., Salameh, B., Sarhan, A. L., & Batran, A. (2023). The factors influencing nurses’ clinical decision-making in emergency department. INQUIRY: The Journal of Health Care Organization, Provision, and Financing, 60, 00469580231152080.

Aboalrob, W., Ayed, A., Malak, M. Z., & Aqtam, I. (2025). Understanding the influence of self-concept on clinical decision-making among nurses: A cross-sectional study. Plos one, 20(8), e0330905.

Methodology

Explain how online survey administration via Questionnaire Star might influence response bias and how this was mitigated.

Provide justification for the choice of cutoff criteria in the data-cleaning process (e.g., 240 seconds, >1 hour), as these could affect data validity.

Measures

The manuscript effectively uses validated instruments but should clarify cultural adaptation processes beyond translation (e.g., pilot testing, expert review panels).

Results and Statistical Reporting

Report confidence intervals for key regression coefficients to enhance transparency.

Discussion and Implications

The discussion effectively interprets dual mediation but occasionally repeats descriptive results. Strengthen the interpretive depth by linking findings to existing frameworks of clinical reasoning and metacognition.

The practical implications should specify how nursing educators can design interventions combining emotional intelligence and self-directed learning, for example, structured reflective practice modules or mentorship-based SDL programs.

Limitations and Future Research

The authors acknowledge cross-sectional limitations; consider also discussing potential social desirability bias and the lack of objective behavioral assessment of decision-making competence.

Recommend conducting a longitudinal or experimental study to verify causal relationships and to test intervention effects.

Ensure consistency in terminology (e.g., “clinical decision-making confidence” vs. “clinical decision-making self-confidence”).

Reviewer #2: The study makes a valuable contribution, and with these revisions, it will be significantly strengthened for publication. Please see the attached, comprehensive review document for detailed comments and suggestions for revision

Reviewer #3: This manuscript addresses an important and timely topic in nursing education and clinical practice by clarifying the mechanisms through which emotional intelligence influences nurses’ clinical decision-making confidence. The study makes a valuable contribution by empirically demonstrating the dual mediating roles of cognitive and relational self-directed learning using a large, well-powered sample and robust statistical modeling.

To further strengthen the theoretical grounding of the study, the authors may consider engaging with Abdallah and Abdallah (2023), which examines self-directed learning as a mechanism for professional competence development in practice-based disciplines. This work could enrich the discussion of how cognitive and relational self-directed learning pathways contribute to confidence and applied decision-making.

Strengths of the manuscript include:

A strong theoretical foundation integrating Self-Determination Theory and Experiential Learning Theory.

Use of validated measurement instruments with excellent psychometric properties.

Rigorous statistical analysis, including SEM and bootstrapped mediation testing.

Clear practical implications for nursing education and professional development.

Suggestions for improvement (minor):

The Discussion section could be slightly streamlined to reduce repetition, particularly where findings are restated multiple times across subsections.

Some interpretations (e.g., department-based differences and experiential explanations) could be presented more concisely to strengthen focus.

While the conclusions are well supported, briefly acknowledging the cross-sectional design when discussing implications would further strengthen methodological transparency.

Reviewer #4: The manuscript must describe a technically sound piece of scientific research with data that supports the conclusions. Experiments must have been conducted rigorously, with appropriate controls, replication, and sample sizes. The conclusions must be drawn appropriately based on the data presented.

Reviewer #5: 1. Mean should be calculated by dividing the total score by the number of items. In this way, for example, when using a 5-point Likert scale, the mean score will range from 1 to 5, which allows for an intuitive interpretation of the magnitude of the mean. Accordingly, all related values and descriptions should be revised to reflect this calculation method.

2. In the Abstract, the phrase “with cognitive pathways demonstrating stronger mediation (33.9%) than relational pathways (24.1%)” is inappropriate, as these pathways were not compared within the same model.

3. The name of the mediating variable is inconsistent with the subsequent description. In Figure 4, it is labeled as learning evaluation, but it appears as relational self-directed learning in the Abstract. This inconsistency should be clarified and corrected.

4. In the Introduction, the research objectives should be presented before the hypotheses.

5. The hypotheses are also problematic. Only one mediation hypothesis is stated in the manuscript; however, two models are tested in the subsequent analyses, each examining a separate mediation effect. If two mediating variables (i.e., treating the two dimensions as separate variables) are assumed, it would be more appropriate to explicitly state two hypotheses, each proposing the existence of a mediation effect. If there are no prior empirical studies examining some of the direct pathways among the variables, this should be explicitly acknowledged and stated in the manuscript.

6.The description of the Self-Rating Scale of Self-Directed Learning (SRSSDL) should be more concise and clearly structured. As currently presented, it is unclear how many dimensions the scale includes, whether it consists of multiple subscales, or how many dimensions are contained within each subscale. This needs to be clarified to avoid confusion.

7.The mean score should be calculated by dividing the total score by the number of items. The analyses should be rerun accordingly, as the values may differ slightly, and the updated values should be reported.

8.The value reported as the total effect is confusing. As it appears to represent the sum of the direct and indirect effects, and all effects are reported as positive, the total effect should not be smaller than either component. The authors should clarify how the total effect was calculated and verify whether the reported values are correct.

Reviewer #6: 1. Theoretical framework and mechanism explanation

Problem: Although the research mentions self-determination theory and experiential learning theory, there is insufficient explanation on how they specifically support the path mechanism of "emotional intelligence → autonomous learning → decision-making confidence". The theoretical foundation of the "relationship path" in the model is weak (such as how interpersonal relationships can be transformed into learning motivation?).

2. Limitations of Causal Inference

Problem: The cross-sectional design cannot confirm causality, but the manuscript implies causality in multiple places (such as "emotional intelligence catalyzes learning behavior"). The full text should clearly emphasize the essential relevance of the research and avoid overinterpreting causality.

3. Insufficient theoretical basis for the restructuring of scale dimensions

The author reorganized the original five factor structure of SRSSDL into two dimensions: cognitive autonomous learning (C-SDL) and social relationship autonomous learning (SR-SDL). Although the Garrison model is cited as theoretical support, has this recombination been tested by confirmatory factor analysis (CFA)? Is the CFA result reported in the article (lines 293-295) for the recombined two factor model or the original five factor model? Suggest the author to provide the comparison results between the two factor model and the original five factor model (such as chi square difference test, AIC/BIC comparison) to demonstrate the rationality of the recombination.

4. Sample representativeness and universality

Question: Women account for 97.56% of the sample, mainly from tertiary hospitals, which limits the generalizability of the conclusion to male nurses and primary healthcare institutions. In addition, women accounted for 97.56% of the sample, while tertiary hospitals accounted for 97.70%. This highly homogeneous sample may limit the extrapolation of research conclusions. The author has mentioned this issue in the Limitations section, but suggests discussing in more detail how these features may affect research findings in the Discussion.

5. Data sources and measurement deviations

Problem: Relying on self-reported data may lead to social expectation bias. Clinical decision-making confidence is a subjective evaluation and is not associated with objective clinical manifestations.

6. The description of the sampling method does not match the actual operation

The author claims to use 'simple random sampling', but the actual process described in the methods section (distributed by nursing department coordinators, recruited during shift changes) is more in line with the characteristics of convenience sampling or cluster sampling. Simple random sampling requires randomly selecting each individual from a complete sampling box. Please clarify the actual sampling process or modify the description of the sampling method.

7. The calculation of the percentage of mediation effect needs to be explained

The author reported that cognitive pathways mediated 33.9% and relational pathways mediated 24.1%, totaling 58% of the mediating effect. However, the direct effects in the Bootstrap analysis table (Table 3) were numerically different between the two models (Model 1: 0.133 vs Model 2: 0.233), suggesting that the two paths may have been estimated separately in different models. Can the author explain whether a complete model has been established that includes both mediation paths simultaneously? If two paths are estimated separately in different models, the simple addition of the two mediation effect percentages may be problematic.

8. Testing for common method bias

There is a risk of common method bias when collecting data using three self-assessment scales at the same time point in the study. Suggest the author report the results of Harman single factor test or other common method bias tests.

9. Complete report of confirmatory factor analysis

The CFA results for three scales were reported in the article, but specific values for factor loadings were missing. Suggest providing complete CFA results in the supplementary materials, including standardized factor loadings and AVE/CR values.

10. In the author contribution statement on line 722, the third author's name is written as "Huan Ye", which is inconsistent with "Juan Ye" in other places. Please verify and unify. The p-value format in some tables is inconsistent (some use "<0.01 * *", some use "<0.01 * *"), it is recommended to use the standard format uniformly.

**********

6. PLOS authors have the option to publish the peer review history of their article (what does this mean?). If published, this will include your full peer review and any attached files.

If you choose “no”, your identity will remain anonymous but your review may still be made public.

**Do you want your identity to be public for this peer review?** For information about this choice, including consent withdrawal, please see our Privacy Policy.

Reviewer #1: No

Reviewer #2: No

Reviewer #3: **Yes:** Asma Abdallah

Reviewer #4: No

Reviewer #5: No

Reviewer #6: No

**********

[NOTE: If reviewer comments were submitted as an attachment file, they will be attached to this email and accessible via the submission site. Please log into your account, locate the manuscript record, and check for the action link "View Attachments". If this link does not appear, there are no attachment files.]

To ensure your figures meet our technical requirements, please review our figure guidelines: https://journals.plos.org/plosone/s/figures

You may also use PLOS’s free figure tool, NAAS, to help you prepare publication quality figures: https://journals.plos.org/plosone/s/figures#loc-tools-for-figure-preparation.

NAAS will assess whether your figures meet our technical requirements by comparing each figure against our figure specifications.

Attachment Submitted filename: Dual Pathways of Self-Directed Learning Review Report.docx

# Author comment: Author response to Decision Letter 1

**DOI:** 10.1371/journal.pone.0351551.r002

30 Mar 2026

Dear Editor and Reviewers,

We sincerely thank the academic editor and all reviewers for their thoughtful and constructive comments on our manuscript. These insights have been invaluable in strengthening the quality and clarity of our work. We have carefully addressed each point raised during the review process and provided a detailed, point-by-point response below. We have included a tracked change (modified sentences marked with red text) of our revised manuscript, and line numbers in this letter refer to the revised version.Detailed responses to reviewers are provided in the attached ‘Response to Reviewers’ file.

Attachment Submitted filename: OK-Point by Point Response to Reviewers.docx

# Peer review: Decision Letter 1

**DOI:** 10.1371/journal.pone.0351551.r003

**Authors:** Maria Nogueira

28 May 2026

Enhancing Nurses' Clinical Decision-Making Confidence Through Dual Pathways of Self-Directed Learning: A Structural Equation Model

PONE-D-25-50160R1

Dear Dr. Juan Ye

We’re pleased to inform you that your manuscript has been judged scientifically suitable for publication and will be formally accepted for publication once it meets all outstanding technical requirements.

Within one week, you’ll receive an e-mail detailing the required amendments. When these have been addressed, you’ll receive a formal acceptance letter and your manuscript will be scheduled for publication.

An invoice will be generated when your article is formally accepted. Please note, if your institution has a publishing partnership with PLOS and your article meets the relevant criteria, all or part of your publication costs will be covered. Please make sure your user information is up-to-date by logging into Editorial Manager at Editorial Manager® and clicking the ‘Update My Information' link at the top of the page. For questions related to billing, please contact billing support.

If your institution or institutions have a press office, please notify them about your upcoming paper to help maximize its impact. If they’ll be preparing press materials, please inform our press team as soon as possible -- no later than 48 hours after receiving the formal acceptance. Your manuscript will remain under strict press embargo until 2 pm Eastern Time on the date of publication. For more information, please contact onepress@plos.org.

Kind regards,

Maria José Nogueira, Ph.D.

Academic Editor

PLOS One

Additional Editor Comments (optional):

The authors made the revisions requested by the reviewers and fully justified all the issues raised.

The manuscript is now ready for acceptance for publication.

Reviewers' comments:

Reviewer's Responses to Questions

**Comments to the Author**

1. If the authors have adequately addressed your comments raised in a previous round of review and you feel that this manuscript is now acceptable for publication, you may indicate that here to bypass the “Comments to the Author” section, enter your conflict of interest statement in the “Confidential to Editor” section, and submit your "Accept" recommendation.

Reviewer #5: All comments have been addressed

Reviewer #7: All comments have been addressed

**********

2. Is the manuscript technically sound, and do the data support the conclusions?

The manuscript must describe a technically sound piece of scientific research with data that supports the conclusions. Experiments must have been conducted rigorously, with appropriate controls, replication, and sample sizes. The conclusions must be drawn appropriately based on the data presented.

Reviewer #5: Yes

Reviewer #7: Yes

**********

3. Has the statistical analysis been performed appropriately and rigorously?

Reviewer #5: Yes

Reviewer #7: Yes

**********

4. Have the authors made all data underlying the findings in their manuscript fully available?

The PLOS Data policy requires authors to make all data underlying the findings described in their manuscript fully available without restriction, with rare exception (please refer to the Data Availability Statement in the manuscript PDF file). The data should be provided as part of the manuscript or its supporting information, or deposited to a public repository. For example, in addition to summary statistics, the data points behind means, medians and variance measures should be available. If there are restrictions on publicly sharing data—e.g. participant privacy or use of data from a third party—those must be specified.

Reviewer #5: Yes

Reviewer #7: Yes

**********

5. Is the manuscript presented in an intelligible fashion and written in standard English?

PLOS ONE does not copyedit accepted manuscripts, so the language in submitted articles must be clear, correct, and unambiguous. Any typographical or grammatical errors should be corrected at revision, so please note any specific errors here.

Reviewer #5: Yes

Reviewer #7: Yes

**********

6. Review Comments to the Author

Please use the space provided to explain your answers to the questions above. You may also include additional comments for the author, including concerns about dual publication, research ethics, or publication ethics. (Please upload your review as an attachment if it exceeds 20,000 characters)

Reviewer #5: The authors have adequately addressed the previous comments, and the manuscript has been substantially improved. The study is well-designed, and the findings are clearly presented and appropriately discussed. Therefore, I recommend to accept the manuscript.

Reviewer #7: I would like to congratulate the authors for addressing a timely and highly relevant topic. It was a pleasure to review this manuscript, which contributes to strengthening research in this field through a large and methodologically sound sample.

The authors responded rigorously and appropriately to the comments previously provided. Overall, the manuscript is clear, well structured, and supported by robust psychometric results, aligning well with the standards of methodological rigor and scientific transparency required by PLOS ONE.

**********

7. PLOS authors have the option to publish the peer review history of their article (what does this mean?). If published, this will include your full peer review and any attached files.

If you choose “no”, your identity will remain anonymous but your review may still be made public.

**Do you want your identity to be public for this peer review?** For information about this choice, including consent withdrawal, please see our Privacy Policy.

Reviewer #5: No

Reviewer #7: No

**********

# Editor report: Acceptance letter

**DOI:** 10.1371/journal.pone.0351551.r004

**Authors:** Maria Nogueira

PONE-D-25-50160R1

PLOS One

Dear Dr. Ye,

I'm pleased to inform you that your manuscript has been deemed suitable for publication in PLOS One. Congratulations! Your manuscript is now being handed over to our production team.

At this stage, our production department will prepare your paper for publication. This includes ensuring the following:

* All references, tables, and figures are properly cited

* All relevant supporting information is included in the manuscript submission,

* There are no issues that prevent the paper from being properly typeset

You will receive further instructions from the production team, including instructions on how to review your proof when it is ready. Please keep in mind that we are working through a large volume of accepted articles, so please give us a few days to review your paper and let you know the next and final steps.

Lastly, if your institution or institutions have a press office, please let them know about your upcoming paper now to help maximize its impact. If they'll be preparing press materials, please inform our press team within the next 48 hours. Your manuscript will remain under strict press embargo until 2 pm Eastern Time on the date of publication. For more information, please contact onepress@plos.org.

You will receive an invoice from PLOS for your publication fee after your manuscript has reached the completed accept phase. If you receive an email requesting payment before acceptance or for any other service, this may be a phishing scheme. Learn how to identify phishing emails and protect your accounts at https://explore.plos.org/phishing.

If we can help with anything else, please email us at customercare@plos.org.

Thank you for submitting your work to PLOS ONE and supporting open access.

Kind regards,

PLOS ONE Editorial Office Staff

on behalf of

Professor Maria José Nogueira

Academic Editor

PLOS One
